# Aldolase A Accelerates Cancer Progression by Modulating mRNA Translation and Protein Biosynthesis via Noncanonical Mechanisms

**DOI:** 10.1002/advs.202302425

**Published:** 2023-07-11

**Authors:** Junjiao Song, Hongquan Li, Yanfang Liu, Xinrong Li, Qili Shi, Qun‐Ying Lei, Weiguo Hu, Shenglin Huang, Zhiao Chen, Xianghuo He

**Affiliations:** ^1^ Fudan University Shanghai Cancer Center and Institutes of Biomedical Sciences Shanghai Medical College Fudan University Shanghai 200032 China; ^2^ Key Laboratory of Breast Cancer in Shanghai Fudan University Shanghai Cancer Center Fudan University Shanghai 200032 China; ^3^ Shanghai Key Laboratory of Radiation Oncology Fudan University Shanghai Cancer Center Fudan University Shanghai 200032 China; ^4^ Collaborative Innovation Center for Cancer Personalized Medicine Nanjing Medical University Nanjing 211166 China

**Keywords:** Aldolase A (ALDOA), eIF4G, hepatocellular carcinoma, IGF2BP1, mRNA translation, protein biosynthesis, insulin‐like growth factor 2 mRNA‐binding protein 1

## Abstract

Aldolase A (ALDOA), a crucial glycolytic enzyme, is often aberrantly expressed in various types of cancer. Although ALDOA has been reported to play additional roles beyond its conventional enzymatic role, its nonmetabolic function and underlying mechanism in cancer progression remain elusive. Here, it is shown that ALDOA promotes liver cancer growth and metastasis by accelerating mRNA translation independent of its catalytic activity. Mechanistically, ALDOA interacted with insulin‐ like growth factor 2 mRNA‐binding protein 1 (IGF2BP1) to facilitate its binding to m^6^A‐modified eIF4G mRNA, thereby increasing eIF4G protein levels and subsequently enhancing overall protein biosynthesis in cells. Importantly, administration of GalNAc‐conjugated siRNA targeting ALDOA effectively slows the tumor growth of orthotopic xenografts. Collectively, these findings uncover a previously unappreciated nonmetabolic function of ALDOA in modulating mRNA translation and highlight the potential of specifically targeting ALDOA as a prospective therapeutic strategy in liver cancer.

## Graphical abstract

Song et al. report a previously unknown nonenzymatic function of ALDOA in HCC progression and establish the direct linkage between ALDOA and mRNA translation in cancer. The newly identified ALDOA‐IGF2BP1‐eIF4G axis provides new insights into the pathogenesis of HCC. Importantly, GalNAc‐siALDOA conjugate opens an avenue to develop a novel strategy of precision therapy for the treatment of HCC.

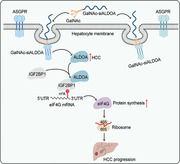

## 1 Introduction

Metabolic reprogramming is a hallmark of malignancy.^[^ ^1^ ^]^ Cancer cells must rewire their metabolism and energy production networks to satisfy the requirements of exponential growth and proliferation and maintain critical cellular processes.^[^ ^2^ ^]^ Metabolic enzymes modulated by critical signaling pathways in cancer cells can fulfill cellular metabolism and growth requirements by performing canonical metabolic functions. However, accumulating evidence has demonstrated that multiple metabolic enzymes can also satisfy the requirements of rapid cancer cell proliferation via noncanonical (“moonlighting”) functions.^[^ ^3^ ^]^ Fructose‐1,6‐bisphosphate (FBP) aldolase is an enzyme that cleaves FBP to dihydroxyacetone phosphate and glyceraldehyde 3‐phosphate in the glycolytic pathway. The aldolase family comprises three isozymes that are structurally very similar: ALDOA, ALDOB, and ALDOC. ALDOA is predominantly expressed in muscle and red blood cells,^[^ ^4^ ^]^ and its aberrant expression drives the pathogenesis of various cancers. ALDOA can promote tumor growth by accelerating glycolysis, and specific ALDOA inhibitors have shown antitumor activity.^[^ ^5^ ^]^ Additionally, several reports have revealed that ALDOA interacts with DNA in the nucleus,^[^ ^6^ ^]^ implying that ALDOA has additional roles beyond its conventional metabolic role. However, the specific functions of ALDOA in cancer progression and the underlying mechanisms remain elusive.

It is increasingly appreciated that translational control not only modulates gene expression but also plays an etiological role in tumorigenesis.^[^ ^7^ ^]^ Typically, mRNA translation includes four steps: initiation, elongation, termination, and ribosome recycling. Translation initiation is the rate‐limiting phase, and dysregulation of translation initiation factors has been documented to cause translational reprogramming, which promotes the synthesis of specific proteins from oncogenes to drive oncogenic transformation and cancer progression.^[^ ^8^ ^]^ Although the connection between the dysregulation of translation machinery components and tumorigenesis has been well established, the upstream factors orchestrating this process remain largely unknown.

Liver cancer is the third leading cause of cancer‐related mortality worldwide. Hepatocellular carcinoma (HCC) accounts for approximately 75%−85% of all liver malignancies.^[^ ^9^ ^]^ Despite tremendous advances in the diagnosis and treatment of HCC in recent decades, the clinical prognosis of HCC remains dismal due to frequent relapse and metastasis after surgical resection,^[^ ^10^ ^]^ and the overall 5‐year survival rate of HCC patients has not significantly improved.^[^ ^11^ ^]^ Therefore, a more comprehensive understanding of the molecular pathogenesis of HCC and more effective therapeutic strategies for HCC treatment are urgently needed.

In this study, we found that ALDOA functions as a translation regulator to facilitate HCC cell growth and metastasis independent of its catalytic activity. Mechanistically, ALDOA interacts with IGF2BP1 to promote the expression of eIF4G by accelerating IGF2BP1 binding to m^6^A‐modified eIF4G mRNA, thereby augmenting oncogenic mRNA translation in HCC. Notably, GalNAc‐siRNA specifically targeting ALDOA demonstrates promising therapeutic efficacy for the treatment of HCC.

## 2 Results

### 2.1 High Expression of ALDOA is Significantly Correlated with Poor Outcomes in Patients with HCC

**Figure 1:**
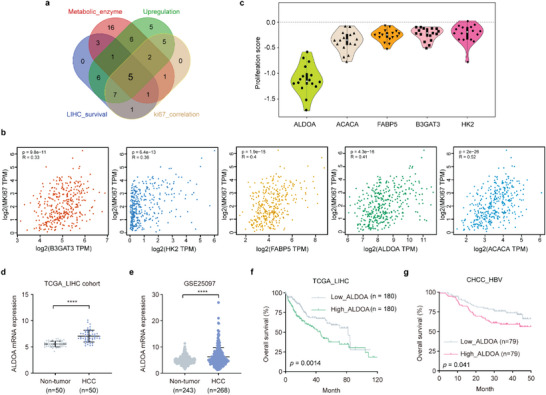
Elevated ALDOA levels correlate with a poor prognosis in HCC patients. a) Venn diagram showing the overlap between the oncogenic metabolic genes identified by CRISPR/Cas9 screening and the genes with elevated levels, which were statistically correlated with poor overall survival and Ki67 staining. b) The correlation between metabolic gene expression and MKI67 expression was determined in the GEPIA dataset. Statistical analysis was performed using Spearman's correlation analysis. c) Significant decreases in proliferation score were observed in 16 liver cancer cell lines after knockout of individual metabolic enzyme genes from project Achilles. d,e) ALDOA mRNA expression between peritumor and tumor tissues in the TCGA_LIHC dataset (Non‐tumor, *n* = 50; Tumor, *n* = 50) (d) and GSE25097 dataset (Non‐tumor, *n* = 243; Tumor, *n* = 268) (e). Data are represented as mean ± SD. Unpaired Student's t‐test. f,g) Kaplan‐Meier overall survival curves for individuals with different ALDOA mRNA expression levels in the TCGA_LIHC dataset (low, *n*  =  180; high, *n* = 180) (f) or CHCC_HBV dataset (low, *n*  =  79; high, *n* = 79) (g) based on univariate Cox regression analysis. ****P < 0.0001.

Our previous CRISPR/Cas9 screening identified 67 metabolism‐related genes as oncogenic candidates involved in HCC tumorigenicity.^[^ ^12^ ^]^ (Figure S1a, Supporting Information). Among these oncogenic candidates, five metabolic enzyme genes, ALDOA, B3GAT3, HK2, FABP5, and ACACA, were highly expressed and significantly correlated with the Ki‐67 level and poor overall survival in HCC (**Figure** **1**a,b). Subsequently, we observed that knockout of these metabolic enzyme genes in liver cancer cells significantly reduced cell proliferation; notably, knockout of ALDOA resulted in the lowest cell proliferation score across 16 liver cancer cell lineages (Figure 1c). Further analyses of ALDOA mRNA levels in The Cancer Genome Atlas (TCGA) and Gene Expression Omnibus  databases demonstrated that increased expression of ALDOA is often observed in HCC patients (Figure 1d,e; Figure S1b, Supporting Information) and high expression of ALDOA is associated with poor survival and pathological characteristics, including TNM stage and tumor size (Figure 1f,g; Figure S1c,d, Supporting Information). These data together indicate that ALDOA may be an important factor in hepatic carcinogenesis.

### 2.2 ALDOA Accelerates HCC Cell Growth and Metastasis In Vitro and In Vivo

**Figure 2:**
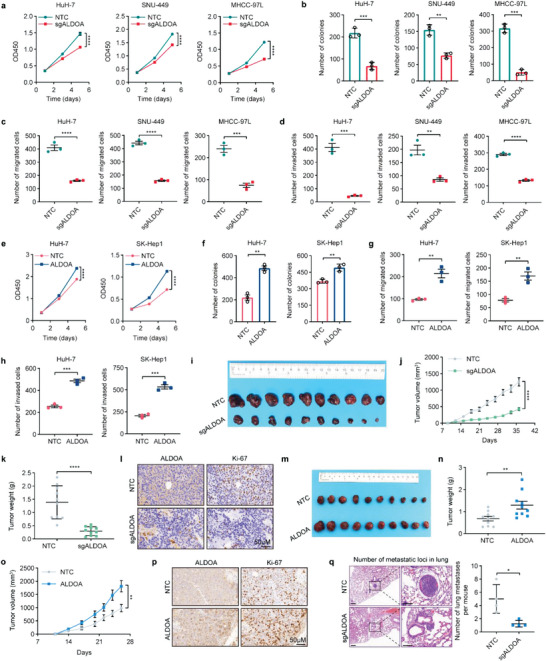
ALDOA promotes HCC cell growth and metastasis in vitro and in vivo. a,b) CCK‐8 assays (a) and colony formation assays (b) for ALDOA knockout HuH‐7, SNU‐449, and MHCC‐97L cells. Data are represented as mean ± SEM (*n* = 3). Two‐way ANOVA with Tukey's multiple comparisons test were performed for (a); unpaired Student's t‐test were performed for (b). c,d) Transwell migration (c) and invasion (d) assays in ALDOA knockout HuH‐7, SNU‐449, and MHCC‐97L cells Data are represented as mean ± SEM (*n* = 3). Unpaired Student's t‐test. e,f) CCK‐8 assays (e) and colony formation assays (f) in ALDOA‐overexpressing HuH‐7 and SK‐Hep1 cells. Data are represented as mean ± SEM (*n* = 3). Two‐way ANOVA with Tukey's multiple comparisons test were performed for (e); unpaired Student's t‐test were performed for (f). g,h) Transwell migration (g) and invasion (h) assays in ALDOA‐overexpressing HuH‐7 and SK‐Hep1 cells. Data are represented as mean ± SEM (*n* = 3). Unpaired Student's t‐test. i) Tumor images for the xenograft mouse model injected with HuH‐7‐NTC or HuH‐7‐sgALDOA cells (*n* = 10). j,k) Quantification of tumor growth (j) and tumor weight (k) following NTC or sgALDOA treatment. Data are represented as mean ± SEM (*n* = 10). Unpaired Student's t‐test. l) Representative IHC staining images of Ki67 from control or ALDOA knockout mouse livers (*n* = 3). Scale bar: 50 µm. m) Images of tumors from the xenograft mouse model injected with HuH‐7‐Vector or HuH‐7‐ALDOA cells (*n* = 10). n,o) Quantification of tumor weight (n) and tumor growth (o) following vector or ALDOA treatment. Data are represented as mean ± SEM (*n* = 10). Unpaired Student's t‐test. p) Representative IHC staining images of Ki67 from Vector‐ or ALDOA‐overexpressing mouse livers (*n* = 3). Scale bar: 50 µm. q) Representative H&E staining images of lung metastatic foci in the orthotopic HCC implantation models. Scale bar: 50 µm. Data are shown as mean ± SEM (*n* = 4). Unpaired Student's t‐tests. *p < 0.05, **p < 0.01, ***p < 0.001, ****p < 0.0001.

To determine the biological functions of ALDOA in HCC, we applied small‐interfering RNAs (siRNAs) against ALDOA to transiently knockdown ALDOA. The results showed that ALDOA knockdown significantly decreased cell viability and colony formation ability in HuH‐7, SNU‐449 and MHCC‐97L cells (Figure S2a–d, Supporting Information). Moreover, the migration and invasion abilities in the ALDOA knockdown group were also dampened, as indicated by transwell assays (Figure S2e,f, Supporting Information). Further CRISPR/Cas9 knockout assays demonstrated that depletion of ALDOA significantly inhibited HCC cell growth, migration and invasion (**Figure** **2**a–d; Figure S3a–c, Supporting Information), whereas forced expression of ALDOA displayed the opposite effects (Figure 2e–h; Figure S3d–f, Supporting Information). Moreover, ALDOA expression significantly affected the glycolytic rate, glucose consumption, and lactate production (Figure S3g–l, Supporting Information). To further delineate the oncogenic role of ALDOA in vivo, we performed xenograft experiments by subcutaneously injecting ALDOA‐knockout or ALDOA‐overexpressing HuH‐7 cells into nude mice. The results showed that ALDOA deficiency significantly retarded tumor growth and reduced tumor volume and weight (Figure 2i–k). Accordingly, staining of the cell proliferation marker Ki67 was reduced in ALDOA‐deficient tumors (Figure 2l). In contrast, ALDOA overexpression strongly accelerated tumor growth (Figure 2m–o), accompanied by higher Ki67 staining (Figure 2p). We next employed an orthotopic xenograft mouse model via intrahepatic injection to evaluate the effects of ALDOA on HCC metastasis. The results showed that knockout of ALDOA dramatically inhibited lung metastasis, as exhibited by HE analyses (Figure 2q). Taken together, these data demonstrate that ALDOA acts as an oncogene during HCC progression.

### 2.3 ALDOA Promotes HCC Progression in a Catalytic‐Activity‐Independent Manner

**Figure 3:**
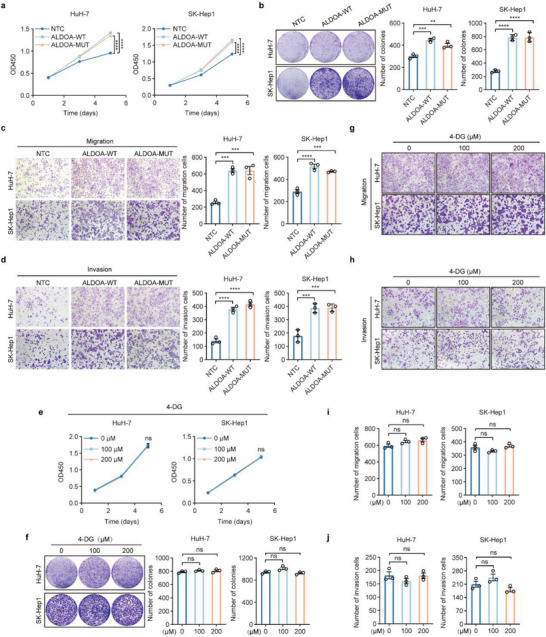
ALDOA enhances HCC progression independent of catalytic activity. a,b) CCK‐8 assays (a) and colony formation assays (b) for ALDOA‐WT‐ or ALDOA‐MUT‐transduced HuH‐7 and SK‐Hep1 cells. Data are represented as mean ± SEM (*n* = 3). Two‐way ANOVA with Tukey's multiple comparisons test were performed for (a); one‐way ANOVA with Dunnett's multiple comparisons test were performed for (b). c,d) Transwell migration (c) and invasion (d) assays for ALDOA‐WT‐ or ALDOA‐MUT‐transduced HuH‐7 and SK‐Hep1 cells. Data are represented as mean ± SEM (*n* = 3). One‐way ANOVA with Dunnett's multiple comparisons test. e,f) CCK‐8 assays (e) and colony formation assays (f) were performed to assess the proliferation of HuH‐7 and SK‐Hep1 cells treated with DMSO or the indicated concentrations of 4‐DG. Data are represented as mean ± SEM (*n* = 3). One‐way ANOVA with Dunnett's multiple comparisons test. g–j) Transwell migration (g, i) and invasion assays (h, j) were performed in HuH‐7 and SK‐Hep1 cells treated with DMSO or the indicated concentrations of 4‐DG. Data are represented as the mean ± SEM (*n* = 3). One‐way ANOVA with Dunnett's multiple comparisons test. ns, not significant, **p < 0.01, ***p < 0.001, ****p < 0.0001.

To explore whether ALDOA‐induced HCC progression is dependent on ALDOA enzymatic activity, we introduced wild‐type ALDOA (ALDOA‐WT) or catalytically inactive ALDOA (ALDOA‐MUT) into endogenous ALDOA‐depleted HuH‐7 or SK‐Hep1 cells. The results showed that forced expression of exogenous ALDOA mutants could not elevate intracellular ALDOA enzymatic activity (Figure S4a, Supporting Information). However, ALDOA mutant overexpression consistently altered HCC cell growth, colony formation, migration and invasion compared with those in the wild‐type group (**Figure** **3**a–d; Figure S4b,c, Supporting Information), indicating that ALDOA plays oncogenic functions independent of its enzymatic activity. To further confirm this finding, we used 4‐DG, which has previously been reported to partially dampen aldolase enzyme activity, in our model.^[^ ^13^ ^]^ Intriguingly, 4‐DG dose‐dependently diminished ALDOA enzymatic activity but failed to impair cellular proliferation (Figure 3e,f; Figure S4d–f, Supporting Information). Consistently, we did not observe any significant alterations in migration and invasion abilities upon 4‐DG treatment (Figure 3g–j). Together, these results suggest that ALDOA serves as an oncogenic regulator in HCC progression independently of its catalytic activity.

### 2.4 ALDOA Accelerates mRNA Translation and Protein Biosynthesis in HCC Cells

**Figure 4:**
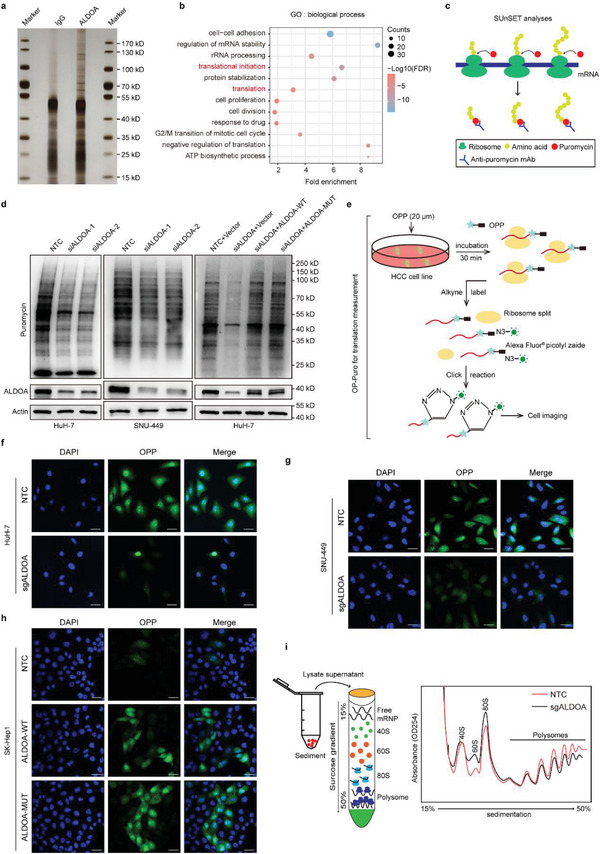
ALDOA accelerates mRNA translation and protein synthesis in HCC. a) Silver staining identifies the specific bands for ALDOA‐IP (versus negative control). b) GO analysis of proteins that interact with ALDOA. c) Schematic diagram of SUnSET. d) The effects of ALDOA on de novo protein synthesis in HuH‐7, and SNU‐449 cells as analyzed by SUnSET assays. e) Schematic diagram of OP‐Puro. f–h) The effects of ALDOA knockout (f,g) or overexpression (h) on protein synthesis in HuH‐7, SNU‐449, and SK‐Hep1 cells as analyzed by OP‐Puro assays (*n* = 3). Scale bar, 50 µm. i) Polysome profiling of 293T cells with ALDOA knockout.

To explore the underlying mechanisms by which ALDOA exerts its oncogenic activity in hepatic carcinogenesis, we conducted coimmunoprecipitation (co‐IP) and subsequent mass spectrometry (MS) analyses to identify the interactome of ALDOA in HCC cells (**Figure** **4**a; Table S1, Supporting Information). Intriguingly, Gene Ontology analyses demonstrated that the ALDOA‐interacting proteins were enriched mostly in translation‐related categories (Figure 4b), suggesting that ALDOA might be involved in translational regulation. To test this hypothesis, we first knocked down ALDOA in HCC cells, subjected the cells to a short burst of puromycin, and then deployed surface sensing of translation (SUnSET) analyses (Figure 4c), an orthodox method that enables monitoring of total cellular translation via detection of puromycin‐labeled polypeptides. Depletion of ALDOA resulted in a remarkable decrease in de novo protein biosynthesis in both HuH‐7 and SNU‐449 cells, whereas reconstituted expression of ALDOA‐WT or ALDOA‐MUT rescued this phenotype (Figure 4d). To further support our observations, we employed O‐propargyl‐puromycin (OP‐Puro) assays by measuring OPP‐tagged polypeptide incorporation (Figure 4e). The results showed that ALDOA‐knockout HuH‐7 and SNU‐449 cells displayed strikingly attenuated global translational activity compared with that of control cells (Figure 4f,g; Figure S5a, Supporting Information), whereas ALDOA‐overexpressing SK‐Hep1 cells showed the opposite effect; interestingly, this trend was more obvious in the ALDOA mutant group than in wild‐type ALDOA‐overexpressing cells (Figure 4h; Figure S5b, Supporting Information). Furthermore, treatment of HCC cells with 4‐DG did not impact protein synthesis, as indicated by SUnSET and OP‐Puro analysis (Figure S5c,d, Supporting Information). These findings suggest that ALDOA is capable of facilitating protein synthesis and that this translation promotion effect is independent of its enzymatic activity. In addition, polysome profiling analysis revealed that knockout of ALDOA resulted in a measurable decrease in polysomes, with a corresponding increase in monosomes (Figure 4i).

### 2.5 ALDOA Interacts with IGF2BP1 to Regulate mRNA Translation in HCC Cells

**Figure 5:**
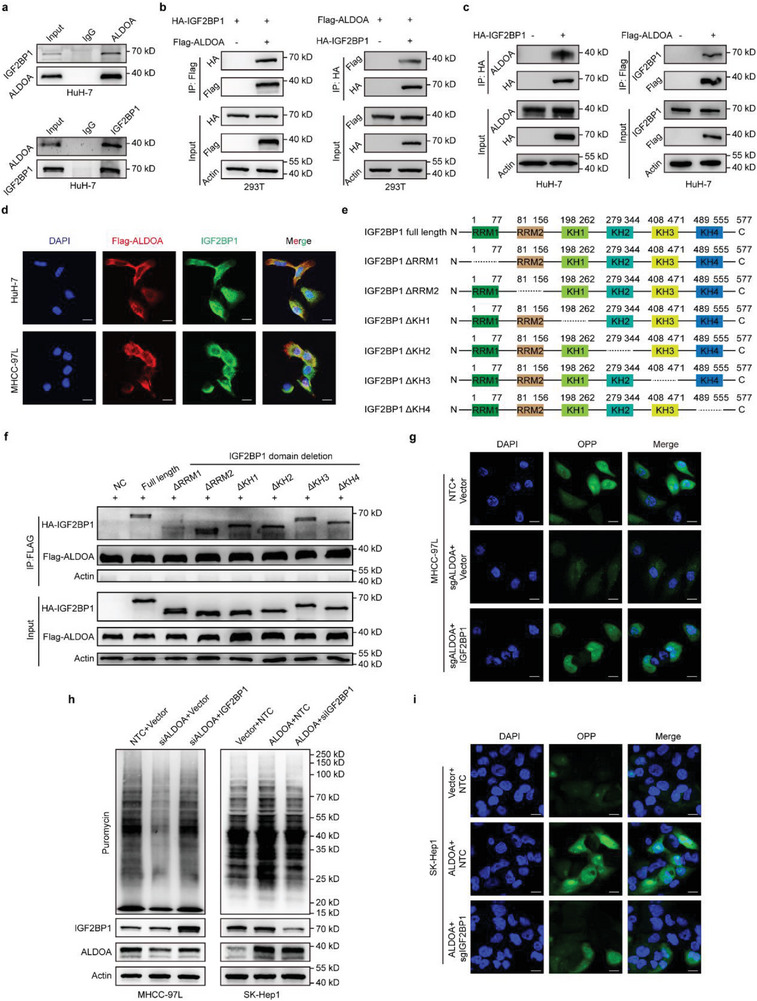
ALDOA interacts with IGF2BP1 to promote mRNA translation. a) Endogenous ALDOA‐IGF2BP1 interactions in HuH‐7 cells detected by co‐IP experiments. b) Exogenous ALDOA‐IGF2BP1 interactions in 293T cells detected by co‐IP experiments. c) The interactions between HA‐tagged IGF2BP1 and endogenous ALDOA (left) or Flag‐tagged ALDOA and endogenous IGF2BP1 were detected by Co‐IP experiments. d) Immunofluorescence of Flag‐tagged ALDOA (red) colocalized with IGF2BP1 (green) in HuH‐7 (upper panels) and MHCC‐97L (lower panels) cells (*n* = 3). Scale bar, 50 µm. e) Schematic for IGF2BP1‐domain‐deletion mutants. f) Flag‐tagged ALDOA Co‐IP with HA‐tagged IGF2BP1 domain‐deletion mutants in 293T cells. g) OP‐Puro analysis showing that the decrease in protein synthesis induced by ALDOA knockout was reversed by IGF2BP1 overexpression in MHCC‐97L cells (*n* = 3). Scale bar, 50 µm. h) SUnSET analyses of MHCC‐97L (left) and SK‐Hep1 (right) cells demonstrating the changes in protein synthesis among the indicated samples. i) OP‐Puro analysis showing that the increase in protein synthesis induced by ALDOA overexpression was attenuated by IGF2BP1 knockout in SK‐Hep1 cells (*n* = 3). Scale bar, 50 µm.

We next sought to determine the exact mechanism by which ALDOA enhances mRNA translation. Among the identified interacting proteins of ALDOA, 14 were classified as translation‐related proteins. Notably, IGF2BP1 exhibited the highest score for ALDOA interaction (Table S2, Supporting Information). Subsequently, in vitro binding assays showed that ALDOA interacted with IGF2BP1 (**Figure** **5**a–c). Moreover, immunofluorescence staining showed that ALDOA and IGF2BP1 colocalized in the cytoplasm (Figure 5d). IGF2BP1 comprises six canonical RNA‐binding domains, including two RNA recognition motifs (RRMs) and four K homology (KH) domains.^[^ ^14^ ^]^ To clarify which domain is responsible for the interaction between ALDOA and IGF2BP1, we constructed a series of deletion mutants. We found that deletion of the RRM1 domain dramatically impaired the ALDOA‐IGF2BP1 interaction (Figure 5e,f), while deletion of the other domains did not affect the binding affinity, indicating that the RRM1 domain is indispensable for the ALDOA‐IGF2BP1 association.

It has recently been reported that IGF2BP1 is involved in mRNA translation.^[^ ^15^ ^]^ To determine whether the translation promoting effect of ALDOA was mediated by IGF2BP1, we first evaluated the functional role of IGF2BP1 in mRNA translation in HCC cells. As expected, knockout of IGF2BP1 markedly decreased nascent protein synthesis in both HuH‐7 and MHCC‐97L cells (Figure S6a,b, Supporting Information), whereas ectopic expression of IGF2BP1 elevated protein synthesis in SK‐Hep1 cells, as demonstrated by OP‐Puro analysis (Figure S6c, Supporting Information). Similar results were also obtained by using a puromycin‐based approach (Figure S6d, Supporting Information). Moreover, overexpression of IGF2BP1 dramatically attenuated the effects of ALDOA depletion on de novo peptide synthesis (Figure 5g,h). Moreover, IGF2BP1 deficiency markedly abolished the increased protein translation induced by ALDOA overexpression (Figure 5h,i). Collectively, these findings demonstrate that ALDOA physically interacts with IGF2BP1 to regulate mRNA translation.

Because ALDOA and IGF2BP1 regulate translation coordinately, we wondered whether IGF2BP1 has a similar function to ALDOA in HCC cells. Not surprisingly, IGF2BP1 knockout substantially suppressed the proliferation, migration and invasion abilities of HCC cells (Figure S7a–e, Supporting Information), whereas IGF2BP1 overexpression had the opposite effects (Figure S7f–j, Supporting Information). In particular, ectopic expression of IGF2BP1 in ALDOA‐silenced HuH‐7 and MHCC‐97L cells partially abolished the reduction in cell proliferation, migration and invasion induced by ALDOA downregulation (Figure S8a–e, Supporting Information). Moreover, depletion of IGF2BP1 in HuH‐7 cells strikingly attenuated the promoting effect of ALDOA overexpression on HCC cell malignancy (Figure S8f–i, Supporting Information). These results clearly show that IGF2BP1 is critical for ALDOA in accelerating HCC progression.

### 2.6 IGF2BP1 Modulates mRNA Translation by Increasing eIF4G Expression in an m^6^A‐Dependent Manner

**Figure 6:**
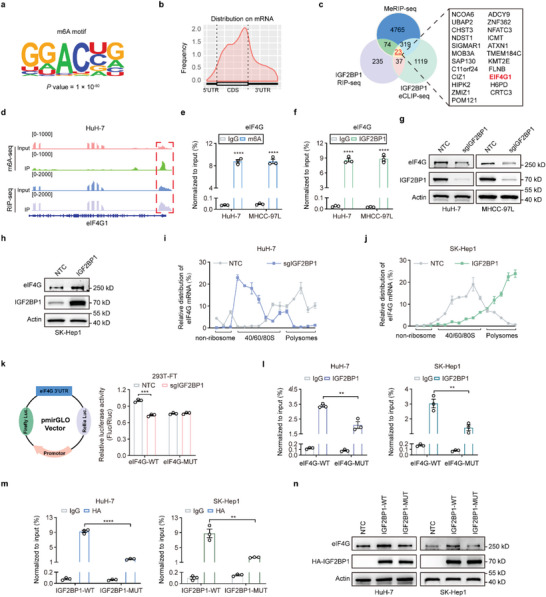
IGF2BP1 modulates mRNA translation by increasing eIF4G expression in an m^6^A‐dependent manner. a) m^6^A motif identified by MEME motif analysis with MeRIP‐seq data in HuH‐7 cells. b) Metagene profiles of m^6^A enrichment across mRNA segments in HuH‐7 cells. c) Venn diagram showing the overlap of genes identified by MeRIP‐seq, IGF2BP1 RIP‐seq, and IGF2BP1 eCLIP‐seq. d) Integrative genomics viewer (IGV) tracks of m^6^A peaks and IGF2BP1‐binding peaks across eIF4G transcript. e) Gene‐specific m^6^A qPCR validation of m^6^A levels in HuH‐7 and MHCC‐97L cells. Data are represented as mean ± SEM (*n* = 3). Unpaired Student's t‐test. f) IGF2BP1 RIP followed by RT‐qPCR detected the interaction between IGF2BP1 and eIF4G mRNA. Data are represented as mean ± SEM (*n* = 3). Unpaired Student's t‐test. g,h) Western blot showing the expression of eIF4G after knockout of IGF2BP1 in HuH‐7 and MHCC‐97L cells (g) or overexpression of IGF2BP1 in SK‐Hep1 cells (h). i,j) Polysome profiling coupled with RT‐qPCR analysis of HuH‐7 cells with IGF2BP1 knockout (i) or overexpression (j) of eIF4G mRNA distribution in different ribosome fractions. k) A schematic presentation of the pMIR‐GLO luciferase reporters containing eIF4G‐3′UTR‐WT (GGAC) or eIF4G‐3′UTR‐MUT (GGCC) (left); luciferase activities of eIF4G‐3′UTR‐WT or eIF4G‐3′UTR‐MUT were measured in 293T cells with or without IGF2BP1 knockout (right). Data are represented as mean ± SEM (*n* = 3). Two‐way ANOVA with Tukey's multiple comparisons test. l) RIP‐qPCR detecting the binding of IGF2BP1 to the transcript of eIF4G‐3′UTR‐WT or eIF4G‐3′UTR‐MUT in HuH‐7 and SK‐Hep1 cells. Data are represented as mean ± SEM (*n* = 3). Two‐way ANOVA with Tukey's multiple comparisons test. m) The association of wild‐type and KH3‐4‐mutated IGF2BP1 with eIF4G mRNA in HuH‐7 and SK‐Hep1 cells as evaluated by RIP‐qPCR. Data are represented as mean ± SEM (*n* = 3). Two‐way ANOVA with Tukey's multiple comparisons test. n) Western blot analysis of eIF4G expression in IGF2BP1‐WT‐ or IGF2BP1‐MUT‐overexpressing HuH‐7 cells. ns, not significant, **p < 0.01, ***p < 0.001, ****p < 0.0001.

Previous studies have reported that IGF2BP1 can recognize and bind with m^6^A‐modified mRNAs and enhance mRNA stability and translation.^[^ ^15^ ^]^ Thus, methylated RNA immunoprecipitation (MeRIP)‐seq and IGF2BP1 RIP‐seq were performed in HuH‐7 cells. MeRIP‐seq identified 9177 m^6^A peaks in 5181 genes (Table S3, Supporting Information). MEME algorithm analysis identified the m^6^A consensus motif (GGAC), implying the successful enrichment of m^6^A‐modified mRNAs (**Figure** **6**a). In accordance with previous studies, m^6^A peaks were primarily enriched in the coding sequence (CDS) and 3′‐untranslated region (3′‐UTR) (Figure 6b; Figure S9a, Supporting Information). The circos plot in Figure S9b (Supporting Information) displays the distribution of methylated m^6^A peaks in the transcriptome of HuH‐7 cells. Moreover, RIP‐seq identified 369 transcripts specifically interacting with IGF2BP1 (fold change≥2, p<0.05) (Table S4, Supporting Information). Next, an integrated approach combining MeRIP‐seq, IGF2BP1 RIP‐seq and enhanced crosslinking and immunoprecipitation (eCLIP)‐seq, (https://www.encodeproject.org/) identified 23 potential direct targets of IGF2BP1 (Figure 6c). Among them, eIF4G, the scaffold component of the eIF4F‐m^7^G cap binding complex, plays a key role in translational regulation. As shown in Figure 6d, m^6^A modifications accumulated across the eIF4G transcript, and the m^6^A peaks coincided well with IGF2BP1 binding sites. Subsequently, MeRIP‐qPCR and RIP assays confirmed both the presence of m^6^A modification and occupancy by IGF2BP1 at eIF4G mRNA in both HuH‐7 and MHCC‐97L cells (Figure 6e,f). Moreover, knockdown of the m^6^A methyltransferase METTL3 strikingly reduced the abundance of m^6^A antibody‐immunoprecipitated eIF4G mRNA (Figure S9c, Supporting Information) and the protein levels of eIF4G in HCC cells (Figure S9d, Supporting Information).

To further determine the effects of IGF2BP1 on m^6^A‐modified eIF4G mRNA, we first knocked out IGF2BP1 in HCC cells and found that IGF2BP1 depletion strikingly reduced the protein abundance of eIF4G (Figure 6g), whereas IGF2BP1 overexpression obviously increased the protein abundance of eIF4G (Figure 6h). Interestingly, the mRNA level of eIF4G remained constant (Figure S9e,f, Supporting Information), indicating that IGF2BP1 might regulate the expression of eIF4G at the post‐RNA level. Subsequently, polysome profiling coupled with qPCR analysis revealed that IGF2BP1 deficiency resulted in a robust shift of eIF4G mRNA to the nonpolysome fractions with attenuation of eIF4G mRNA levels in the translation fractions (Figure 6i). In contrast, IGF2BP1 overexpression led to a shift of eIF4G mRNA to polysome fractions with increased eIF4G mRNA levels in the translation fractions (Figure 6j). Collectively, these results indicate that IGF2BP1 can increase the translation output of eIF4G in HCC.

Next, we explored the underlying mechanism by which IGF2BP1 exerts the m^6^A‐mediated posttranscriptional regulation of eIF4G in HCC cells. We inserted the wild‐type (WT) eIF4G‐3′UTR sequence or a mutant counterpart whose putative m^6^A sites were mutated into a firefly luciferase reporter (Figure S9g, Supporting Information). As expected, the luciferase activity of the eIF4G‐WT reporter was significantly reduced upon IGF2BP1 knockout, but the mutant groups were resistant to the effect of IGF2BP1 depletion (Figure 6k). Consistently, RIP‐qPCR assay showed strong binding of IGF2BP1 with eIF4G‐WT plasmids but much weaker binding of IGF2BP1 with eIF4G‐MUT in HuH‐7 and SK‐Hep1 cells (Figure 6l; Figure S9h, Supporting Information). These results suggested that m^6^A modifications in the eIF4G‐3′UTR are essential for the binding of IGF2BP1 to eIF4G mRNA and for IGF2BP1‐mediated regulation of eIF4G expression. IGF2BP1 recognizes and binds with m^6^A sites mainly through the KH3‐4 di‐domain. Mutations of GxxG to GEEG in the KH3‐4 di‐domain can dampen the binding capacity of IGF2BP1 with m^6^A‐modified mRNA.^[^ ^15^ ^]^ Thus, an HA‐tagged mutant IGF2BP1 construct (IGF2BP1‐MUT) with conversion of GxxG to GEEG in the KH3‐4 di‐domain was established to abolish the m^6^A binding pocket (Figure S9i, Supporting Information). We found that the interaction between IGF2BP1‐MUT and eIF4G mRNA was significantly attenuated (Figure 6m; Figure S9j, Supporting Information). Consistently, the increased expression of eIF4G in the IGF2BP1‐WT group was eliminated in the IGF2BP1‐MUT group due to impaired m^6^A reader activity (Figure 6n; Figure S9k, Supporting Information). Taken together, these data indicated that IGF2BP1 directly binds to m^6^A‐modified eIF4G mRNA and promotes eIF4G expression in an m^6^A‐dependent manner.

### 2.7 ALDOA Facilitates IGF2BP1 Binding to eIF4G mRNA and Increases its Translational Output

**Figure 7:**
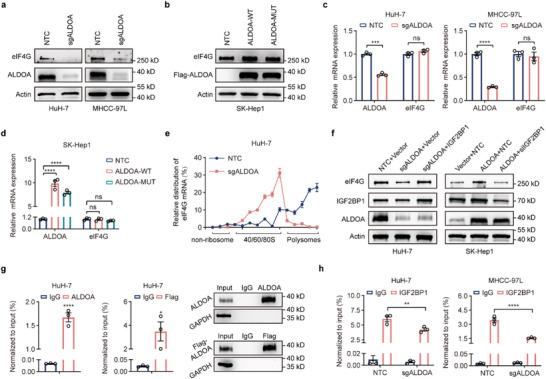
ALDOA facilitates IGF2BP1 binding to eIF4G mRNA in HCC cells. a,b) Western blot analysis of eIF4G expression in ALDOA knockout HuH‐7 and MHCC‐97L cells (a) or ALDOA‐WT‐ or ALDOA‐MUT‐overexpressing SK‐Hep1 cells (b). c,d) RT‐qPCR analysis of eIF4G expression in ALDOA knockout HuH‐7 and MHCC‐97L cells (c) or ALDOA‐WT‐ or ALDOA‐MUT‐overexpressing SK‐Hep1 cells (d). Data are represented as mean ± SEM (*n* = 3). Unpaired Student's t‐test were performed for (c); one‐way ANOVA with Dunnett's multiple comparisons test were performed for (d). e) Polysome–qPCR analysis of eIF4G expression in ALDOA knockout HuH‐7 cells. f) Levels of eIF4G in ALDOA knockout (left) or ALDOA‐overexpressing (right) cells restored with IGF2BP1. g) RIP‐qPCR detecting the binding of ALDOA to the transcript of eIF4G in HuH‐7 cells. Data are represented as mean ± SEM (*n* = 3). Unpaired Student's t‐tests. h) RIP‐qPCR detecting the binding of IGF2BP1 to the transcript of eIF4G upon ALDOA knockout in HuH‐7 and MHCC‐97L cells. Data are represented as mean ± SEM (*n* = 3). Two‐way ANOVA with Tukey's multiple comparisons test. ns, not significant, *p < 0.05, **p < 0.01, ***p < 0.001, ****p < 0.0001.

Given that IGF2BP1 directly regulates the protein abundance of eIF4G, we next analyzed whether ALDOA has a similar effect as IGF2BP1 in HCC cells. Consistent with previous observations, knockout of ALDOA dramatically reduced the protein level of eIF4G (**Figure** **7**a), whereas overexpression of either ALDOA‐WT or ALDOA‐MUT elevated the protein level of eIF4G (Figure 7b). However, neither ALDOA depletion nor ALDOA overexpression had a clear impact on the mRNA level of eIF4G (Figure 7c,d). Subsequently, polysome profiling coupled with qPCR revealed that the relative distribution of eIF4G mRNA was shifted from polysome to subpolysome fractions in the ALDOA‐depleted HuH‐7 cells compared to the corresponding controls (Figure 7e), illustrating that ALDOA is capable of promoting the translation of eIF4G. Furthermore, ectopic expression of IGF2BP1 successfully rescued the inhibitory effect of ALDOA deficiency on eIF4G, whereas knockdown of IGF2BP1 suppressed the upregulation of eIF4G induced by ALDOA overexpression (Figure 7f).

Next, we investigated how ALDOA coordinates with IGF2BP1 to modulate eIF4G translation. Generally, translational regulators directly bind to the mRNA of target genes to modulate mRNA translation. We therefore conducted a RIP assay and found that eIF4G mRNA was significantly enriched in the ALDOA‐IP sample compared with the IgG‐IP sample (Figure 7g). A comparable phenomenon was also observed in FLAG‐tagged ALDOA overexpressing HuH‐7 cells utilizing the same strategy (Figure 7g). Most importantly, knockout of ALDOA dramatically impaired the capability of IGF2BP1 to bind eIF4G mRNA (Figure 7h). Collectively, our results demonstrate that the effect of ALDOA on eIF4G translation is IGF2BP1‐dependent and that ALDOA augments eIF4G translation by enhancing IGF2BP1 binding to eIF4G mRNA. Additionally, as shown in Figure S10a–c (Supporting Information), knockout of eIF4G resulted in repression of HCC cell viability and colony formation capabilities. Moreover, treating HCC cells with SBI‐756, a well‐characterized eIF4G‐specific inhibitor, phenocopied eIF4G knockout by reducing cell proliferation (Figure S10d,e, Supporting Information). Furthermore, ectopic expression of eIF4G attenuated the decreases in cell viability and colony formation ability in ALDOA‐knockout cells (Figure S10f,g, Supporting Information), whereas treating ALDOA‐overexpressing HCC cells with SBI‐756 to impede protein biosynthesis dramatically dampened the enhancement of cell viability, colony formation ability and invasion capability induced by ALDOA overexpression (Figure S11, Supporting Information), indicating that eIF4G is a functional target of ALDOA in HCC cells.

### 2.8 GalNAc‐siALDOA Administration Effectively Slows the Growth of Orthotopic Tumor Xenografts In Vivo

**Figure 8:**
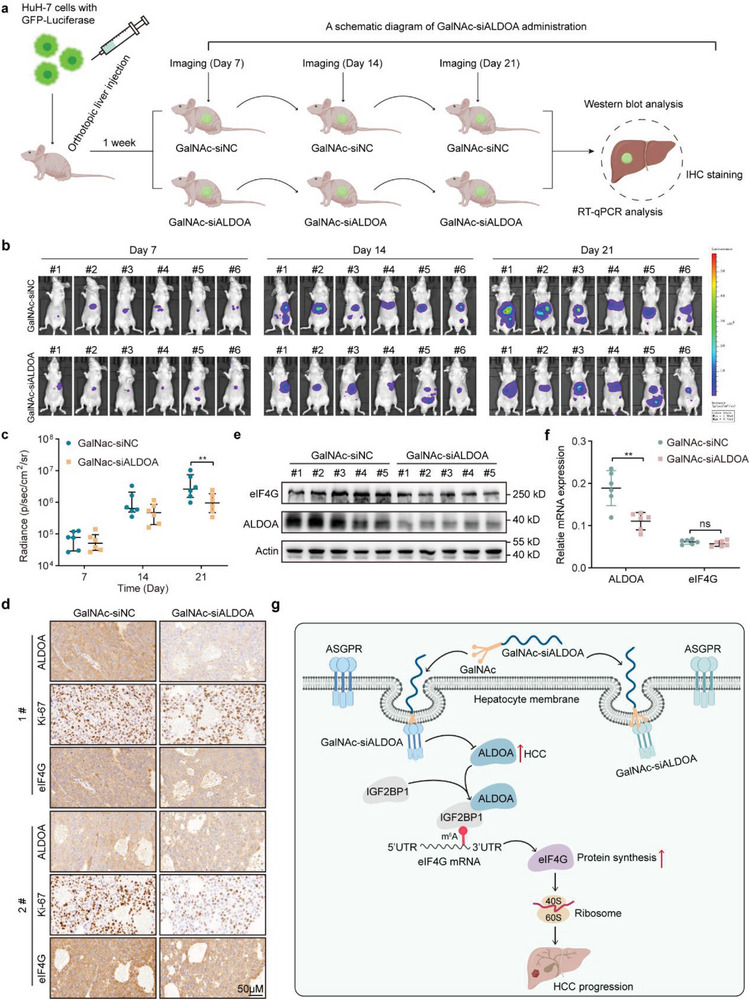
Administration of GalNAc‐siALDOA effectively suppresses the tumor growth of orthotopic xenografts in vivo. a) A schematic diagram of GalNAc‐siALDOA administration. b) GFP‐Luciferase‐transduced HuH‐7 cells were intrahepatically implanted into BALB/c nude mice, and mice were treated with GalNAc‐siNC or GalNAc‐siALDOA (5 mg kg^−1^) once a week for three consecutive weeks. Tumor growth was monitored by assessing luciferase activity (n = 6). c) Scatter plot exhibits the average radiance of signaling intensity. Data are represented as mean ± interquartile range (*n* = 6). Two‐way ANOVA with Sidak's multiple comparisons test. d) Representative images of IHC staining of ALDOA, Ki67, and eIF4G from the GalNAc‐siNC‐ or GalNAc‐siALDOA‐treated group (*n* = 3). Scale bar, 50 µm. e) Western blot detecting the expression of ALDOA and eIF4G in the GalNAc‐siNC‐ or GalNAc‐siALDOA‐ treated group (n = 5). f) RT‐qPCR detecting the expression of ALDOA and eIF4G in the GalNAc‐siNC‐ or GalNAc‐siALDOA‐treated group. Data are represented as mean ± SD (*n* = 6). Unpaired Student's t‐test. g) A schematic demonstrating ALDOA interacts with IGF2BP1 to promote the expression of eIF4G by accelerating IGF2BP1 binding to m^6^A‐modified eIF4G mRNA, thereby augmenting oncogenic mRNA translation in HCC. (g) was created with BioRender.com with an academic license. ns, not significant, **p < 0.01.

siRNA therapeutics have developed rapidly in recent years. The drug performance of siRNA largely depends on the siRNA design, chemical modification and delivery platform.^[^ ^16^ ^]^ Notably, the application of a synthetic multivalent *N*‐acetylgalactosamine (GalNAc) ligand that specifically binds to the asialoglycoprotein receptor (ASGPR) to stimulate the uptake of siRNA into hepatocytes has transformed the field.^[^ ^17^ ^]^ ALDOA is highly expressed in HCC but has relatively low expression in normal hepatocytes, as it is a muscle‐type aldolase,^[^ ^5^, ^18^ ^]^ which prompted us to hypothesize that GalNAc‐siALDOA administration is a prospective therapeutic strategy for targeting HCC. To validate this hypothesis, we orthotopically injected HuH‐7 cells with luciferase labeling into the livers of nude mice. The mice were subcutaneously administered GalNAc‐siALDOA or the corresponding control GalNAc‐siNC (*n* = 6 mice/group) at a dose of 5 mg kg^−1^ for three doses. Tumor growth was subsequently monitored for up to three weeks by a bioluminescence in vivo imaging system (IVIS) (**Figure** **8**a). Our data showed that treatment with GalNAc‐siALDOA significantly impaired the growth of tumor xenografts in mice (Figure 8b,c). Accordingly, the staining rate of the cell proliferation marker Ki67 was reduced in the GalNAc‐siALDOA treatment group (Figure 8d). As expected, the protein levels of ALDOA and eIF4G were markedly reduced after administration of GalNAc‐siALDOA (Figure 8e). However, the mRNA levels of eIF4G remained constant (Figure 8f). This observation is consistent with our aforementioned hypothesis that ALDOA regulates the expression of eIF4G at the translational level. Taken together, these findings suggest that GalNAc‐siALDOA administration is a promising therapeutic approach for targeting HCC with high ALDOA expression.

## 3 Discussion

A hallmark of cancer is the great versatility of metabolic enzymes in cancer cells during tumor progression and evolution, which is reflected by the ability of cancer cells to exert canonical and noncanonical functions.^[^ ^19^ ^]^ In this study, we observed that high expression of ALDOA correlates with a poor prognosis in patients with HCC and is functionally critical for liver cancer malignancy. Importantly, ALDOA interacts with IGF2BP1 to facilitate its binding to m^6^A‐modified eIF4G mRNA, increasing eIF4G protein abundance in a catalytic activity‐independent manner and thereby promoting overall mRNA translation in HCC (Figure 8g). These observations uncover a previously uncharacterized property of ALDOA, a classical glycolytic enzyme that has been extensively investigated, in the regulation of mRNA translation and protein biosynthesis.

Enhanced glycolysis is a hallmark of malignancy and is elicited by overactivated oncogenes, ultimately resulting in dysregulated expression of glycolytic enzymes in tumors.^[^ ^20^ ^]^ Accumulating evidence has recently revealed that multiple metabolic enzymes exhibit unexpected activities apart from their canonical roles in supporting malignant transformation. For instance, ALDOB can interact with glucose‐6‐phosphate dehydrogenase,^[^ ^21^ ^]^ phosphorylated AKT,^[^ ^22^ ^]^ and insulin receptor^[^ ^23^ ^]^ to suppress HCC cell growth. In AML, hexokinase 2 (HK2) can localize to the nucleus, and interact with nuclear proteins to maintain stemness.^[^ ^24^ ^]^ As a critical catalytic enzyme in the glycolytic pathway, ALDOA participates in cancer development through diverse mechanisms in the tumor microenvironment. ALDOA has been reported to facilitate cancer cell proliferation and metastasis by accelerating glycolysis.^[^ ^5^ ^]^ In addition, several studies have demonstrated that ALDOA modulates instrumental cellular activities by impacting the cell cytoskeleton,^[^ ^25^ ^]^ oncogenic signaling pathways,^[^ ^26^ ^]^ and interacting partners^[^ ^13^ ^]^ during carcinogenesis. In this study, we found that ALDOA can interact with IGF2BP1, which serves as an m^6^A reader to stabilize and promote eIF4G protein synthesis, thereby enhancing oncogenic translation in the development and progression of HCC. Our work uncovers a previously uncharacterized and unexpected molecular mechanism for ALDOA in human cancer, which strengthens our understanding of the essential nonmetabolic functions and novel molecular action of ALDOA in tumorigenesis.

IGF2BP1 acts as an m^6^A reader that recognizes the consensus GGAC sequence and targets a variety of mRNA transcripts to control the fate of mRNAs by regulating mRNA subcellular localization, stability, and translation.^[^ ^15^, ^27^ ^]^ To date, only a few target transcripts have been identified as direct downstream effectors of the reader protein IGF2BP1 in cancer. Here, we provide the first evidence that IGF2BP1 interacts with eIF4G mRNA and recognizes m^6^A sites in the eIF4G‐3′UTR, thus increasing the translation output of eIF4G mRNA in an m^6^A‐dependent manner. IGF2BP1 consists of six characteristic RNA‐binding domains, including two RNA‐recognition motifs (RRMs) in the N‐terminal region, and four distinct KH domains in the C‐terminal region.^[^ ^28^ ^]^ The KH3‐4 di‐domain of IGF2BP1 is critical for its binding to m^6^A‐modified mRNAs.^[^ ^15^ ^]^ Here, we found that the KH3‐4 di‐domain of IGF2BP1 was also necessary for its interaction with m^6^A‐modified eIF4G. Most importantly, IGF2BP1 was able to control the overall translation machinery by modulating the protein abundance of a single target, eIF4G mRNA. Control of mRNA translation plays crucial roles in cell growth, survival, and tumorigenesis.^[^ ^29^ ^]^ Aberrations in translational control frequently occur in human cancers, often through activating key signaling pathways, i.e., c‐Myc, PI3K‐mTOR, and Ras‐MAPK pathways, but also through ectopic expression of translation initiation factors.^[^ ^8^, ^30^ ^]^ However, the direct impact of metabolic genes on protein translation is still poorly understood. Here, we revealed that the metabolic enzyme ALDOA plays a positive role in facilitating eIF4G‐dependent protein translation in hepatic carcinogenesis. Notably, ALDOA interacts with and recruits IGF2BP1 to the 3′UTR of eIF4G mRNA; therefore, these factors jointly regulate its translational output and consequently enhance overall protein biosynthesis. These findings reveal that an overactivated ALDOA‐IGF2BP1‐eIF4G axis promotes translation and cancer progression, providing a theoretical target for the development of small molecules in the clinic.

GalNAc is a carbohydrate moiety that binds to ASGPR with high affinity and facilitates the uptake of siRNAs into liver hepatocytes via clathrin‐mediated endocytosis.^[^ ^31^ ^]^ GalNAc‐conjugated siRNAs can specifically target hepatocyte‐deregulated genes for the treatment of liver diseases, making them attractive therapeutic tools. GIVLAARI (givosiran), the first GalNAc‐siRNA drug targeting hepatocyte aminolevulinate synthase 1, has been approved by the FDA for the treatment of acute hepatic porphyria.^[^ ^32^ ^]^ Our previous work deploying a GalNAc‐siRNA conjugate targeting HK2 obtained unprecedented anti‐HCC efficacy in vivo.^[^ ^12^ ^]^ ALDOA is absent or rarely expressed in normal hepatocytes, as it is a muscle‐type aldolase, but it is highly expressed in HCC; therefore, it is a promising therapeutic target for HCC. Here, we used orthotopic HCC xenografts to show that administration of GalNAc‐ALDOA effectively slowed HCC cell growth in vivo; concomitantly, the protein level of eIF4G was decreased. Future investigations are warranted to characterize whether GalNAc‐siALDOA is efficient in HCC patient‐derived xenograft and organoid models before developing GalNAc‐siALDOA drugs for HCC in the clinic.

In conclusion, this study reveals a previously unknown nonenzymatic function of ALDOA in liver cancer growth and metastasis and establishes a direct link between metabolic genes and overall mRNA translational control in cancer. ALDOA interacts with and recruits the RBP IGF2BP1 to m^6^A‐modified eIF4G mRNA, which coordinately enhances eIF4G protein abundance and is involved in eIF4G‐initiated protein biosynthesis. The newly defined ALDOA‐IGF2BP1‐eIF4G axis deepens our understanding of mRNA translation control and cancer pathogenesis. Administration of GalNAc‐siALDOA specifically suppresses the growth of orthotopic xenografts, which provides a promising therapeutic strategy for the treatment of HCC.

## 4 Experimental Section

### Nascent Protein Synthesis Assay

De novo protein synthesis was quantified by OP‐Puro analysis. In brief, HCC cells were treated with OPP at 37 °C for 30 min followed by fixation with 3.7% formaldehyde for 15 min. After washing with PBS three times, cells were permeabilized with 0.25% Triton X‐100 for 15 min. Next, a Click‐iT Plus OPP Alexa Fluor 488 Protein Synthesis Assay kit (Thermo Fisher Scientific, USA) was employed to detect the fluorescent labeling of nascent peptides with OPP incorporation according to the manufacturer's instructions.

### Surface Sensing of Translation (SUnSET) Assay

The SUnSET assay was performed as previously described.^[^ ^33^ ^]^ Briefly, HCC cells were incubated with 10 µg mL^−1^ puromycin for 15 min, followed by chasing for 50 min to confirm that puromycin‐labeled proteins were efficiently detected. Cells were then collected and lysed in RIPA lysis buffer (Beyotime, Shanghai, China). Cell lysates were analyzed by western blotting and probed with an anti‐puromycin antibody (Millipore, USA).

### Polysome Profiling

Cells at ≈90% confluence were pretreated with 100 µg mL^−1^ cycloheximide (CHX) at 37 °C for 5 min. Cells were then scraped and incubated with 800 µL polysome cell extraction buffer containing 10 × 10^−3^ m NaCl, 10 × 10^−3^ m MgCl_2_, 10 × 10^−3^ m Tris–HCl pH 7.5, 1% Triton X‐100, 1% sodium deoxycholate, 0.2 U µL^−1^ RNase inhibitor, 1 × 10^−3^ m DTT and 0.1 mg mL^−1^ cycloheximide. After centrifugation at 16000 × g for 10 min at 4 °C, the supernatants were collected and loaded onto 10%−50% sucrose gradients followed by ultracentrifugation at 274 000 g in a Beckman SW41 rotor for 1 h 40 min at 4 °C. The samples were fractionated by a density gradient fractionation system (Biocomp, Canada), and the absorbance at 254 nm was detected. Collected fractions were subjected to RT‐qPCR analysis.

### MeRIP‐qPCR and MeRIP‐seq (m^6^A‐seq)

MeRIP‐qPCR was performed using the riboMeRIP m^6^A Transcriptome Profiling Kit (RiboBio, Guangzhou) according to the manufacturer's instructions with slight modifications. Briefly, total RNA was collected and digested by DNase I and then fragmented into ≈100 bp‐long fragments using RNA Fragmentation Buffer followed by incubation at 70 °C for 10 min. Subsequently, 0.5 m EDTA was added to each sample to stop the fragmentation reaction. The RNA beads were then incubated with fragmented RNA for 15 min on ice. After standard ethanol precipitation twice, the supernatants were collected. The m^6^A antibody (5 µg) was preincubated with Protein G in IP buffer at room temperature for 1.5 h. Next, the fragmented RNA was incubated with m^6^A antibody‐bead mixture at 4 °C overnight. Enrichment of m^6^A‐containing mRNA was used for RT‐qPCR analysis or RNA‐seq library construction. The primers designed for MeRIP‐qPCR analysis are listed in Table S5 (Supporting Information). The library was prepared by the smart‐seq method. Both the input samples and the m^6^A IP samples were subjected to 150‐bp paired‐end sequencing on an Illumina NovaSeq 6000 sequencer. MeRIP‐seq and sequent data analyses were mainly supported by Epibiotek (Guangzhou, China).

### RNA Immunoprecipitation and High‐Throughput Sequencing (RIP‐seq)

HCC cells were collected and suspended in IP lysis buffer containing 1 U µL^−1^ RNase inhibitor (Vazyme, Nanjing, China). The supernatant was then harvested by centrifugation at 10 000 × g for 15 min after incubation on ice for 30 min. The indicated IP grade antibodies coupled to protein G Dynabeads were added to the lysate followed by incubation overnight at 4 °C. After washing six times with NT2 buffer, the coprecipitated RNAs and input were extracted by TRIzol reagent and analyzed by RT‐qPCR. For sequencing, the KAPA RNA HyperPrep Kit (Roche, USA) was used to remove rRNAs, and cDNA libraries were generated by employing the NEBNext Ultra Directional RNA Library Prep kit (New England Biolabs, USA) and sequenced on the Illumina HiSeq 3000 platform.

### Animal Experiments

For xenograft experiments, 2 × 10^6^ HuH‐7 cells stably overexpressing ALDOA or expressing sgALDOA were subcutaneously injected into BALB/c nude mice (male, 4–6 weeks of age). Tumor size was measured three times per week, and tumor volume was calculated as follows: volume (mm^3^) = 1/2 × length × width^2^. Seven weeks later, the mice were sacrificed, and tumor tissues were immediately isolated for protein extraction or fixed in 10% formalin for immunohistochemistry.

For metastasis experiments, 1 × 10^7^ NTC or sgALDOA HuH‐7 cells were directly injected into the livers of BALB/c nude mice (male, 5–6 weeks old). Eight weeks after injection, the mice were sacrificed, and the lungs were preserved for molecular, biochemical and histological analysis.

For the GalNAc‐siALDOA therapy study, 5 × 10^6^ luciferase tagged HuH‐7 cells were injected into BALB/c nude mice to establish an orthotopic liver tumor xenograft. One week after injection, the mice were subjected to imaging with an IVIS Lumina LT Series III Bioluminescence and Fluorescence imaging system (PerkinElmer, Waltham, MA, USA) after administering 150 mg kg^−1^ D‐luciferin substrate (Yeasen, Shanghai, China). Living Image software was utilized to quantify the bioluminescent signals. Based on the intensity of bioluminescence, the mice were randomly divided into two groups, and GalNAc‐siNC or GalNAc‐siALDOA (5 mg kg^−1^) was given subcutaneously once a week. At the experimental endpoint, tumor tissues were isolated for further analysis.

All animal experiments were performed in accordance with protocols approved by the Institutional Animal Care and Use Committee of Fudan University (permission number: FUSCC‐IACUC‐2022287), Shanghai, China.

### Statistical Analyses

Each experiment was performed with at least three independent replicates. Data were represented as either mean ± SD or mean ± SEM as stated. *P* values were determined by using unpaired Student's t‐test or one‐way analysis of variance (ANOVA) as indicated in corresponding figure legends. *p* < 0.05 was considered significant. All statistical analyses were performed using GraphPad Prism 8.0 software (GraphPad Software, CA, USA).

## Conflict of Interest

The authors declare no conflict of interest.

## Author Contributions

J.S., H.L., and Y.L. contributed equally to this work. X.H., J.S., and Z.C. conceived and designed the study. J.S., H.L., Y.L., and X.L. performed the experiments. J.S., H.L., Y.L., Z.C., Q.S., and S.H. processed the data. J.S., Z.C., Q.L., W.H., and X.H. wrote and revised the manuscript. All authors read and approved the final manuscript.

## Supporting information

Supporting Information

Supplemental Table 1

Supplemental Table 2

Supplemental Table 3

Supplemental Table 4

Supplemental Table 5

## Data Availability

The data that support the findings of this study are available on request from the corresponding author. The data are not publicly available due to privacy or ethical restrictions.
