# Supplementary material for: Aldolase A Accelerates Cancer Progression by Modulating mRNA Translation and Protein Biosynthesis via Noncanonical Mechanisms
Source: Adv Sci (Weinh). 2023 Jul 11;10(26):2302425. doi: 10.1002/advs.202302425 (PMC10502857; doi:10.1002/advs.202302425)
Supplement: Supplementary file 1 — Supporting Information [file ADVS-10-2302425-s002.pdf]

ADVANCED SCIENCE
Open Access

Supporting Information

for *Adv. Sci.*, DOI 10.1002/advs.202302425

Aldolase A Accelerates Cancer Progression by Modulating mRNA Translation and Protein Biosynthesis via Noncanonical Mechanisms

Junjiao Song, Hongquan Li, Yanfang Liu, Xinrong Li, Qili Shi, Qun-Ying Lei, Weiguo Hu, Shenglin Huang, Zhiao Chen and Xianghuo He**

Supporting Information

Aldolase A Accelerates Cancer Progression by Modulating mRNA Translation and Protein Biosynthesis via Noncanonical Mechanisms

Junjiao Song[1], Hongquan Li[1], Yanfang Liu[1], Xinrong Li[1], Qili Shi[1], Qun-Ying Lei[1], Weiguo Hu[1], Shenglin Huang[1], Zhiao Chen[1,2,3,*], Xianghuo He[1,2,3,4,*]

[1]Fudan University Shanghai Cancer Center and Institutes of Biomedical Sciences, Shanghai Medical College, Fudan University, Shanghai 200032, China

[2]Key Laboratory of Breast Cancer in Shanghai, Fudan University Shanghai Cancer Center, Fudan University, Shanghai 200032, China.

[3]Shanghai Key Laboratory of Radiation Oncology, Fudan University Shanghai Cancer Center, Fudan University, Shanghai, 200032, China

[4]Collaborative Innovation Center for Cancer Personalized Medicine, Nanjing Medical University, Nanjing 211166, China

These authors contributed equally to this work: Junjiao Song, Hongquan Li, Yanfang Liu.

[*] **Corresponding Authors:**

Xianghuo He, Email: xhhe@fudan.edu.cn or Zhiao Chen, Email: zachen@fudan.edu.cn, Fudan University Shanghai Cancer Center and Institutes of Biomedical Sciences; Shanghai Medical College, Fudan University, 302 Rm., 7# Bldg., 270 Dong An Road, Shanghai 200032, China. Tel: 86-21-34777329; Fax: 86-21-64172585.

Supplementary Materials and Methods

Cell lines and cell culture

HuH-7, SNU-449, SK-Hep1, MHCC-97L, and HEK-293T cells were cultured in Dulbecco's modified Eagle's medium (DMEM) (BasalMedia, Shanghai, China) supplemented with 10% fetal bovine serum (FBS) (HyClone, Logan, UT, USA), 100 μg mL^{-1} penicillin (Gibco), and 100 μg mL^{-1} streptomycin (Gibco) at 37 °C in 5% CO_2. All cell lines used in this study has been validated by STR profiling and routinely detected mycoplasma using specific primers via RT-qPCR (**Table S5**).

RNA interference and Plasmid construction

Transient RNA interference was performed using small interfering RNA (siRNA) synthesized by RiboBio (Guangzhou, China). The sgRNA for ALDOA, IGF2BP1 and EIF4G knockout was designed deploying online tool (https://zlab.bio/guide-design-resources), and cloned into lentiCRISPR v2 vector (Addgene) for CRISPR editing. The siRNA and sgRNA sequences were listed in **Table S6**. The open reading frames (ORFs) of ALDOA and IGF2BP1 were cloned into the lentivirus expression vector pCDH-CMV-MCS-EF1-Puro (pCDH) (Addgene). pCDNA3.1-Flag-EIF4G was purchased from WZ Biosciences Inc. (Jinan, China). For exogeneous Co-IP analysis, ALDOA and IGF2BP1 were cloned into the PCMV-N-Flag and PCMV-N-HA vector (Beyotime, Shanghai, China), respectively. The primers were listed in **Table S5**. All constructs were verified by DNA sequencing.

RNA extraction and RT-qPCR analysis

Total RNA from HCC cell lines, animal tumor tissues as well as clinical tissue specimens was isolated using TRIzol (Life Technologies, CA, USA) following the manufacturer's instructions. Subsequently, 5 µg of total RNA were reverse-transcribed using the PrimeScript RT Reagent kit (TaKaRa, Tokyo, Japan) at 37°C for 20 min followed by 85°C for 30s and stored at -20°C. Real-time qPCR was performed using

SYBR® Green Pro Taq HS (Accurate Biology, Changsha, China) in 7900 Real-Time PCR apparatus (Applied Biosystems, USA). ACTB was used as internal control for normalization. Primers for RT-qPCR are shown in **Table S5**.

Western blot analysis

Cells were lysed in RIPA buffer containing protease inhibitor cocktail and PMSF (Beyotime, Shanghai, China). Protein extracts were then separated by 10% SDS-PAGE, transferred to NC membranes followed by blocked with 5% non-milk powder in TBST. After probing with specific primary antibodies overnight at 4 °C, the membranes were washed with TBST, and then incubated in HRP-conjugated secondary antibodies in room temperature for 40 min. The membranes were visualized using the Omni-ECL™ Reagent kit (Epizyme, Shanghai, China). Antibodies used in this study are provided in **Table S7**.

Immunoprecipitation and Mass Spectrometry

Approximately 2×10^7 cells were collected and then lysed in IP lysis buffer (50×10^{-3} M Tris-HCl pH7.5, 2×10^{-3} M $MgCl_2$, 150×10^{-3} M NaCl, 10% glycerol, 0.5% NP-40, 1× proteinase inhibitor cocktail and PMSF) for 30 min on ice. After centrifugation at 4 °C, 12000 g for 10 min, the supernatants were incubated with indicated antibodies and Protein G magnetic beads overnight at 4 °C. The following day, the lysate beads mixture was washed three times with NT2 buffer (50×10^{-3} M Tris-HCl pH 7.5, 200×10^{-3} M NaCl, 1×10^{-3} M $MgCl_2$, 0.05% NP-40) and diluted in 1 × SDS loading buffer followed by western blot analysis. ALDOA-interacting proteins were identified by mass spectrometry analysis (Shanghai Applied Protein Technology, Shanghai, China).

Cell proliferation, migration and invasion assay

Cell Counting Kit-8 (CCK-8) (MCE, Shanghai, China) and colony formation assay were used to assess cell proliferation ability. For CCK-8 assay, 2000 cells were seeded in 96-well plate. After attachment, cells were replaced with fresh medium containing 10% CCK-8 reagent, and cell viability was measured at 450 nm absorbance for indicated time points. For colony formation assay, 3000 cells were seeded in six-well plate and cultured for 7-10 days. The colonies were subsequently stained with 1% crystal violet solution and quantified using ImageJ analysis software.

The migration assay was performed using transwell chamber system (8 μm pores, Corning, USA). Briefly, 3×10^4 cells with different treatments were seeded in the upper chamber of an insert with 200 μl serum-free medium in 24-well plate, 600 μl DMEM medium containing 20% FBS were added to the lower chamber. After

incubation at 37 °C for 24 h, the migrated cells were stained with 1% crystal violet dye and counted in four randomly selected fields with Inverted Microscopes (10×) (Olympus, Japan). The invasion assay was implemented similarly, with coating the filters with Matrigel.

Immunohistochemistry

Immunohistochemistry was conducted as the following procedure. In short, paraffin embedded slides were dewaxed in xylene followed by rehydrated in graded ethanol and distilled water sequentially. Slides were immersed in 0.3% hydrogen peroxide for 10 min to block endogenous peroxidase activity, and blocked with 5% BSA for 20 min at room temperature. After probed with indicated primary antibodies overnight at 4 °C, the slides were subsequently washed with PBS triplicates. Next, sections were incubated with HRP-conjugated secondary antibodies at 37 °C for 30 min. Slides were then exhibited by DAB staining. The antibodies used for IHC staining are displayed in **Table S7**.

Dual-luciferase reporter assay

The 3'UTR sequence of eIF4G mRNA was inserted into the downstream of the Firefly luciferase coding region of the dual-luciferase reporter vector pmirGLO (Promega, USA) to construct the pmirGLO-eIF4G-3'UTR WT or pmirGLO-eIF4G-3'UTR Mut plasmid, respectively. IGF2BP1 knockout or corresponding control cells were seeded in 24-well plate and transfected with 500 ng

pmirGLO-eIF4G-3'UTR WT, pmirGLO-eIF4G-3'UTR Mut or pmirGLO control plasmids. After 48 h, cells were harvested and the luciferase activity was measured employing Dual Luciferase Reporter Assay Kit (Promega, USA). The sequences inserted into the pmirGLO vector are displayed in **Table S5**.

Immunofluorescence staining

HCC cells with indicated treatments were seeded in eight-well chamber slides followed by fixation with 4% paraformaldehyde for 15 min at room temperature. After washing with PBS twice, cells were permeabilized with 0.25% Triton X-100 in PBS for 15 min, and then blocked in Immunol Staining Blocking Buffer (Beyotime, Shanghai, China) for 30 min. After that, cells were incubated with primary antibodies overnight at 4 °C. Next day, cells were incubated with fluorescence-labeled secondary antibodies in PBS at room temperature for 1 h. Nuclei were stained by DAPI (Beyotime, Shanghai, China). Images were acquired with confocal microscopy (Leica, Mannheim, Germany).

Aldolase activity assay

Aldolase activity was assessed employing Aldolase Activity Colorimetric Assay Kit (BioVision, USA) following the supplier's instructions. Briefly, 1×10^6 HCC cells were lysed in 100 μl ice cold Aldolase Assay Buffer. After centrifugation at 10000 g for 5 min, the supernatant was collected, and added into 96-well plate with reaction mix (aldolase assay buffer, aldolase enzyme mix, aldolase developer and aldolase

substrate). Absorbance at 450 nm was measured after reaction for 30 min at 37°C.

Measurement of ECAR, glucose consumption and lactate production

The glycolytic rate was examined by using XF Glycolysis Stress Test Kit (Agilent, USA). In brief, HCC cells (initial density of 2×10^4 cells/well) with indicated treatments were seeded into XF96-well plate. Next day, cells were changed with XF DMEM Base Medium followed by sequential injection of 10×10^{-3} M glucose, 1×10^{-6} M oligomycin and 50×10^{-3} M 2-deoxyglucose (2-DG). After incubated in a CO_2-free incubator for 1 h at 37 °C, cells were subjected to extracellular acidification rate analysis with Seahorse XF-96 Wave software.

Glucose uptake assay was conducted using Glucose Colorimetric Assay Kit (BioVision, USA). Briefly, cells were seeded in six-well plate, the culture medium was collected and mixed with glucose assay buffer coupled to glucose reaction buffer in a 96-well plate. After incubated at room temperature for 30 min, the absorbance was measured at 450 nm. For lactate production assay, cells were collected and resuspended in lactate assay buffer followed by centrifugation at 12000 g for 5 min at 4 °C. The concentration of lactate was then measured employing CheKine™ Micro Lactate Assay Kit (Abbkine, Wuhan, Hubei) according to the manufacture's procedure.

Bioinformatics analysis

The single gene loss-of-functions were calculated from Project Achilles. We collected

the proliferation score of liver cancer cell lines for the individual metabolic enzyme genes knockout condition from CRISPR knockout screens (DepMap Portal: https://depmap.org/portal/). The gene-level scores were generated using CERES, and normalized per cell line so that nonessential genes have a median score of 0 and independently identified common essentials have a median score of -1. Negative scores imply cell growth inhibition following gene knockout.[1] ALDOA mRNA level was analyzed based on TCGA_LIHC dataset (http://www.tcga-data.nci.nih.gov) and NCBI GEO database (GSE25097, GSE144269, GSE45436, GSE76427, and GSE64041). The survival analysis of ALDOA in TCGA_LIHC and CHCC_HBV was performed by GraphPad Prism 8.0 software. GO enrichment analysis was performed using online tools (DAVID: https://david.ncifcrf.gov/). IGF2BP1 eCLIP-seq data was download from ENCODE database (https://www.encodeproject.org/).

References

[1] R. M. Meyers, J. G. Bryan, J. M. McFarland, B. A. Weir, A. E. Sizemore, H. Xu, N. V. Dharia, P. G. Montgomery, G. S. Cowley, S. Pantel, A. Goodale, Y. Lee, L. D. Ali, G. Jiang, R. Lubonja, W. F. Harrington, M. Strickland, T. Wu, D. C. Hawes, V. A. Zhivich, M. R. Wyatt, Z. Kalani, J. J. Chang, M. Okamoto, K. Stegmaier, T. R. Golub, J. S. Boehm, F. Vazquez, D. E. Root, W. C. Hahn, A. Tsherniak, *Nat Genet* **2017**, *49*, 1779.

Supplementary Figures

Supplementary Figure 1.

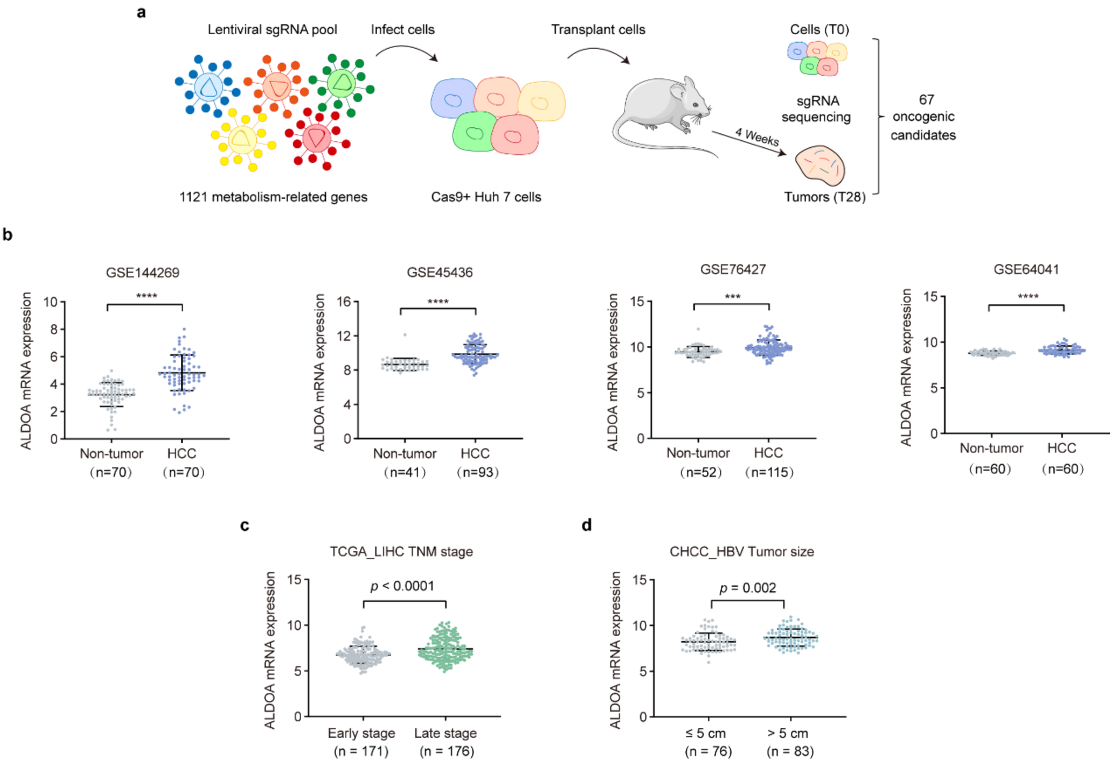

Figure S1. ALDOA is highly expressed in HCC and positively correlated with HCC malignancies.

(a) A schematic diagram of genome-scale CRISPR/Cas9 screening. **(b)** ALDOA mRNA expression between peritumor and tumor tissues was analyzed from GEO (GSE144269, GSE45436, GSE76427, and GSE64041) dataset. **(c)** Correlation between ALDOA mRNA expression and TNM stage in patients with HCC from TCGA dataset (early stage, n = 171; late stage, n = 176). **(d)** Correlation between ALDOA mRNA expression and tumor size in patients with HCC from CHCC_HBV dataset (≤ 5 cm, n = 76; > 5 cm, n = 83). Data are represented as mean ± SEM.

Unpaired Student’s t-tests were performed for **(b-d)**. ***$p < 0.001$, ****$p < 0.0001$.

Supplementary Figure 2.

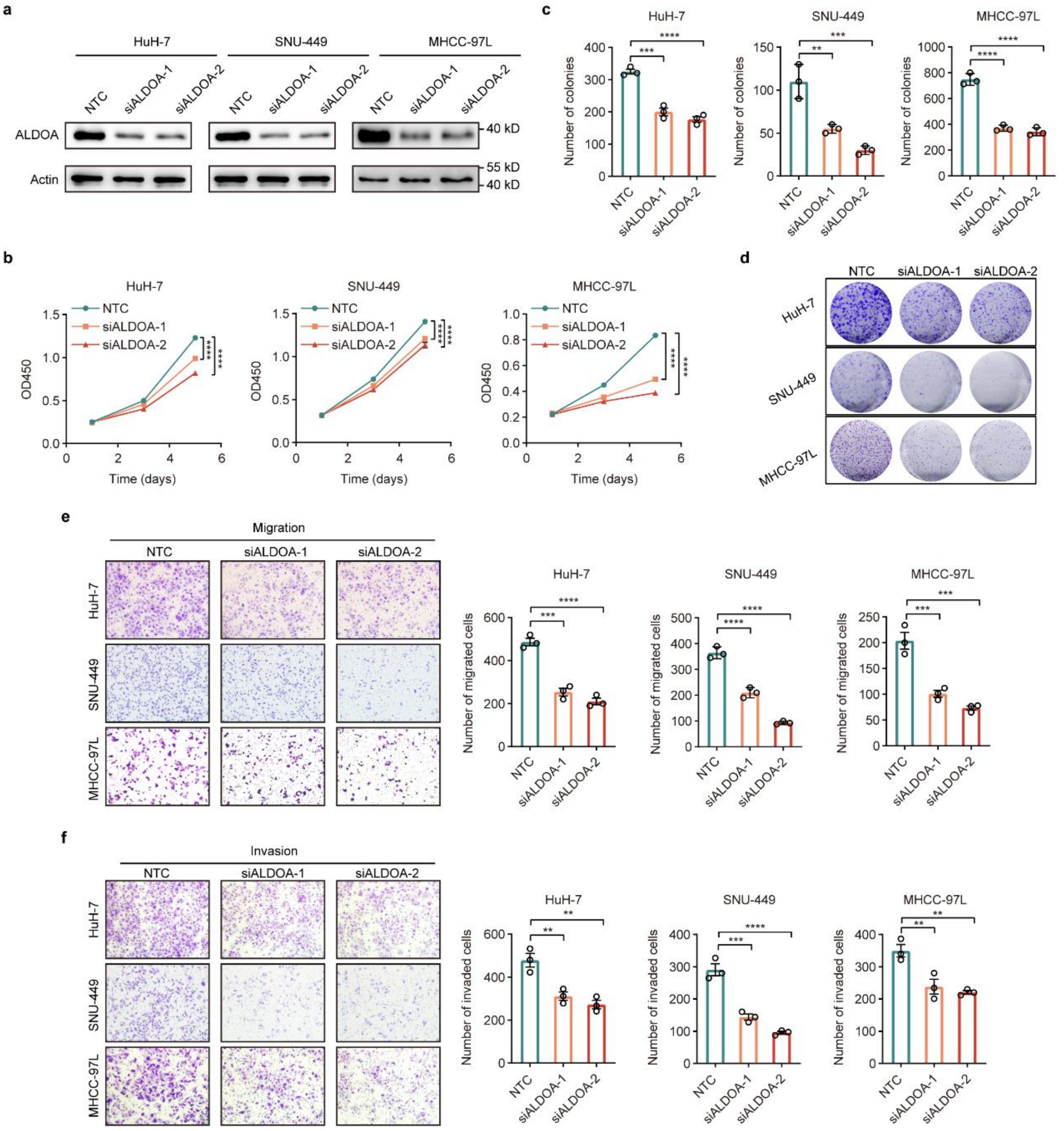

Figure S2. ALDOA accelerates HCC cell proliferation, migration, and invasion

(a) Western blot analysis of ALDOA expression in NTC or siALDOA-transfected HuH-7, SNU-449, and MHCC-97L cells. **(b)** Effects of ALDOA knockdown on cell viability in HuH-7, SNU-449, and MHCC-97L cells. Data are represented as mean ± SEM ($n = 3$). Two-way ANOVA with Tukey's multiple comparisons test. **(c-d)**

Colony formation assay: statistical histogram of colonies from three replicates **(c)** and representative colony images of HuH-7, SNU-449, and MHCC-97L cells with or without ALDOA knockdown **(d)**. Data are represented as mean ± SEM (n = 3). One-way ANOVA with Dunnett's multiple comparisons test. **(e)** Transwell migration assay: representative migration images of HuH-7, SNU-449, and MHCC-97L cells with or without ALDOA knockdown (left) and statistical histogram of migration cells from three replicates (right). Data are represented as mean ± SEM (n = 3). One-way ANOVA with Dunnett's multiple comparisons test. **(f)** Transwell invasion assay: representative invasion images of HuH-7, SNU-449, and MHCC-97L cells with or without ALDOA knockdown (left) and statistical histogram of invasion cells from three replicates (right). Data are represented as mean ± SEM (n = 3). One-way ANOVA with Dunnett's multiple comparisons test. **p < 0.01, ***p < 0.001, ****p < 0.0001.

Supplementary Figure 3.

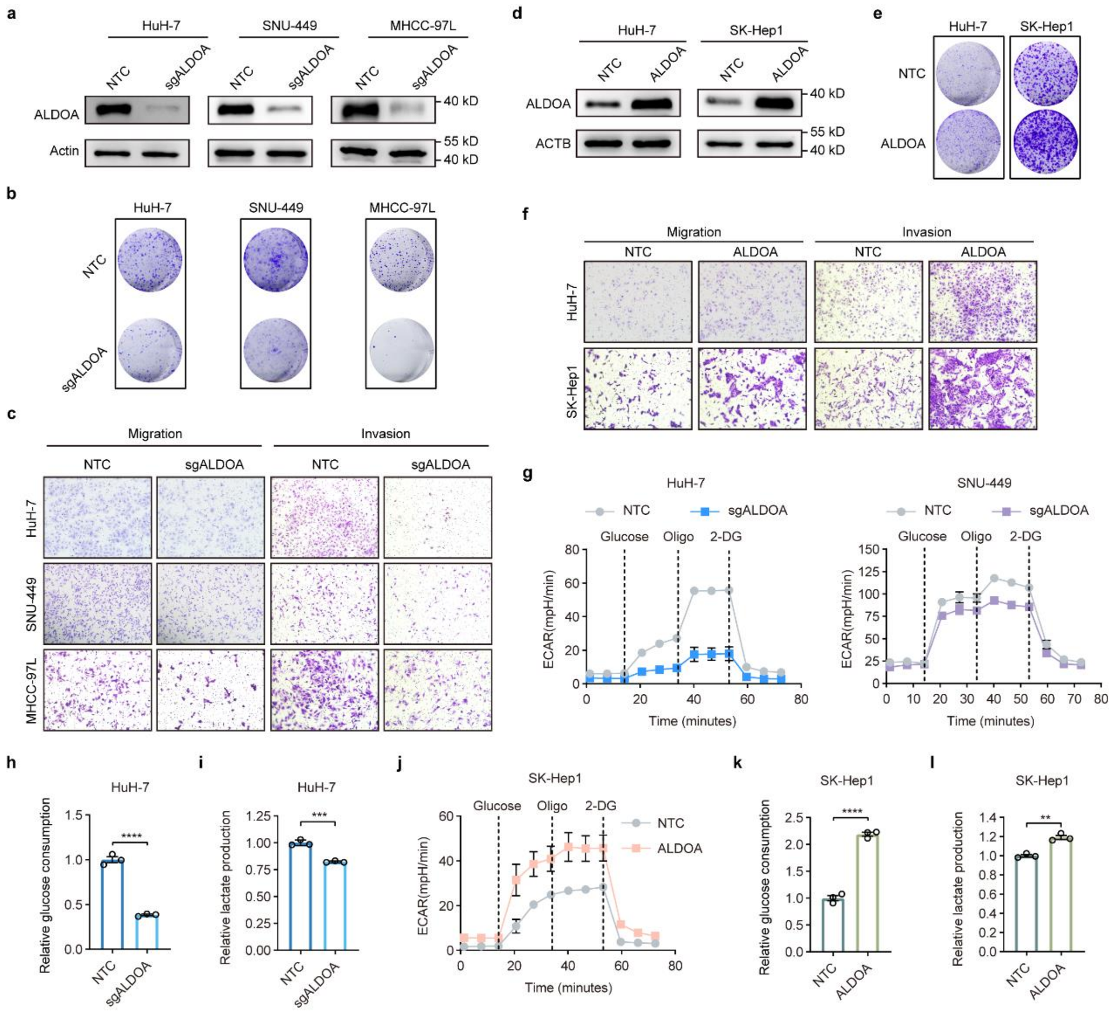

Figure S3. ALDOA promotes HCC cell proliferation, migration, invasion and glycolysis.

(a) Western blot analysis of ALDOA expression in control or ALDOA-knockout HuH-7, SNU-449, and MHCC-97L cells. **(b)** Representative colony images of HuH-7, SNU-449, and MHCC-97L cells with or without ALDOA knockout (*n* = 3). **(c)** Representative migration and invasion images of HuH-7, SNU-449, and MHCC-97L cells with or without ALDOA knockout (*n* = 3). **(d)** Western blot analysis of ALDOA expression in control or ALDOA-overexpressing HuH-7 and SK-Hep1 cells. **(e)**

Representative colony images of HuH-7 and SK-Hep1 cells with or without ALDOA overexpression (n = 3). **(f)** Representative migration and invasion images of HuH-7 and SK-Hep1 cells with or without ALDOA overexpression (n = 3). **(g-i)** Glycolytic rate (ECAR) **(g)**, glucose consumption **(h)**, and lactate production **(i)** changes in HCC cells upon ALDOA knockout. Data are represented as mean ± SEM (n = 3). Unpaired Student's t-test. **(j-l)** Glycolytic rate (ECAR) **(j)**, glucose consumption **(k)**, and lactate production **(l)** changes in HCC cells upon ALDOA overexpression. Data are represented as mean ± SEM (n = 3). Unpaired Student's t-test. **$p < 0.01$, ***$p < 0.001$, ****$p < 0.0001$.

Supplementary Figure 4.

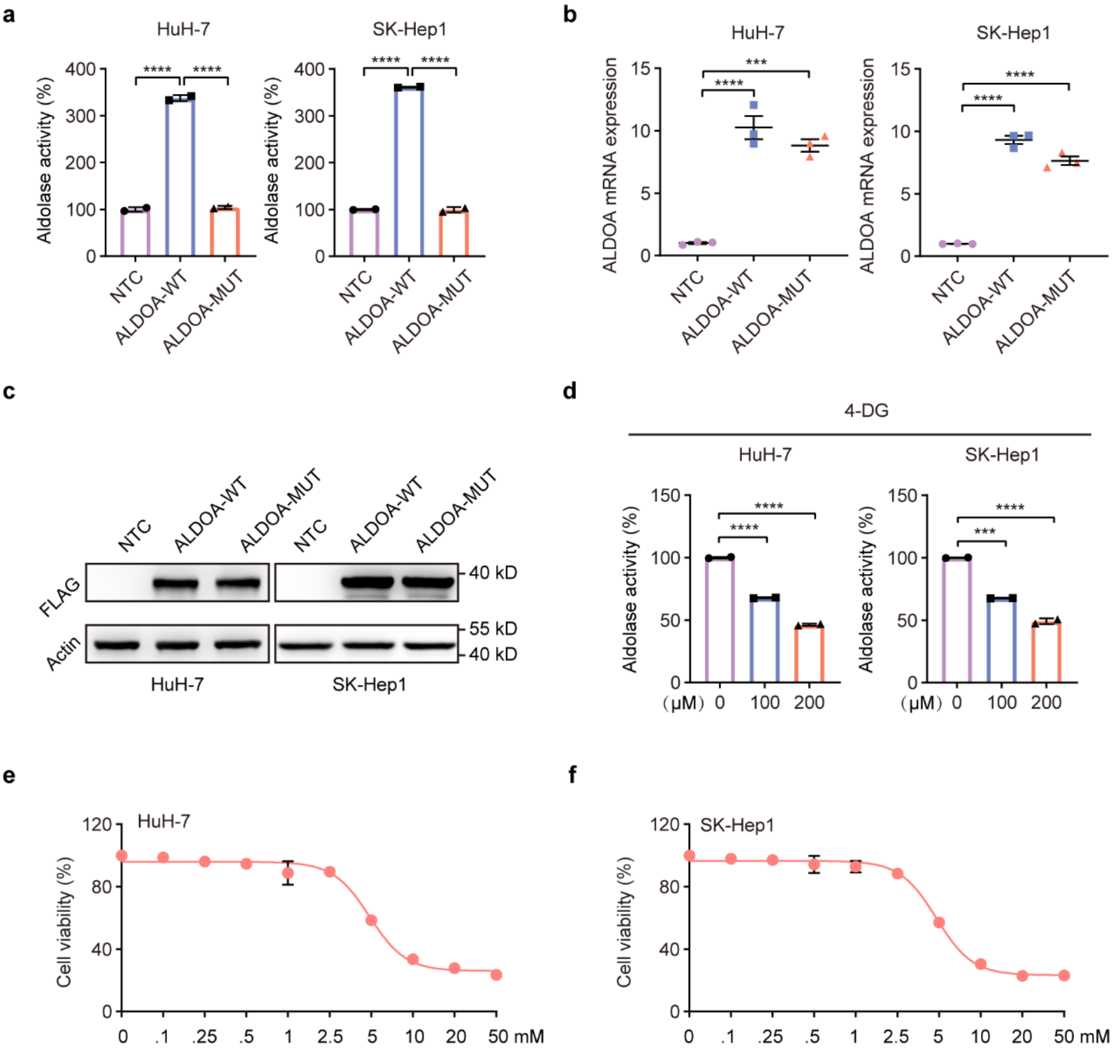

Figure S4. Aldolase activity of ALDOA mutants and 4-DG-treated in HCC cell model.

(a) Aldolase activity in ALDOA-WT or ALDOA-MUT overexpressing HuH-7 and SK-Hep1 cells. Data are represented as mean ± SD (*n* = 2). One-way ANOVA with Dunnett's multiple comparisons test. **(b)** RT-qPCR of ALDOA expression in ALDOA-WT or ALDOA-MUT-transduced HuH-7 and SK-Hep1 cells. Data are represented as mean ± SEM (*n* = 3). One-way ANOVA with Dunnett's multiple comparisons test. **(c)** Western blot of ALDOA expression in ALDOA-WT or

ALDOA-MUT-transduced HuH-7 and SK-Hep1 cells. **(d)** Aldolase activity in 4-DG treated HuH-7 and SK-Hep1 cells. Data are represented as mean ± SD (n = 2). One-way ANOVA with Dunnett's multiple comparisons test. **(e-f)** Growth curves of HuH-7 **(e)** and SK-Hep1 **(f)** cells with different concentrations of 4-DG treatment. ***$p < 0.001$, ****$p < 0.0001$.

Supplementary Figure 5.

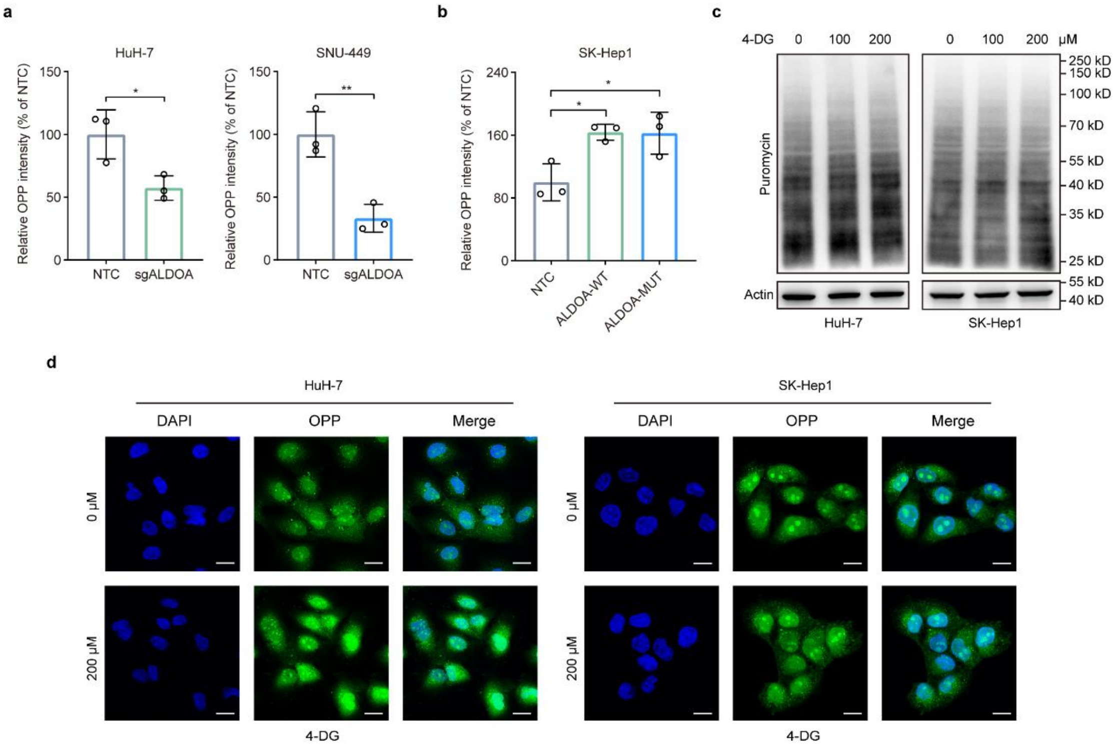

Figure S5. ALDOA promotes translation as indicated by SUnSET and OPP assays.

(a-b) Quantification of OPP signal intensity in ALDOA knockout (**a**) or overexpressing (**b**) HCC cells. Data are presented as mean ± SD (n = 3). Unpaired Student's t-test were performed for (**a**); One-way ANOVA with Dunnett's multiple comparisons test were performed for (**b**). (**c**) The effects of 4-DG on de novo protein synthesis were evaluated by SUnSET assay. **(d)** The effects of 4-DG on protein synthesis were evaluated by OP-Puro assay. Scale bar, 50 μm. *$p < 0.05$, **$p < 0.01$.

Supplementary Figure 6.

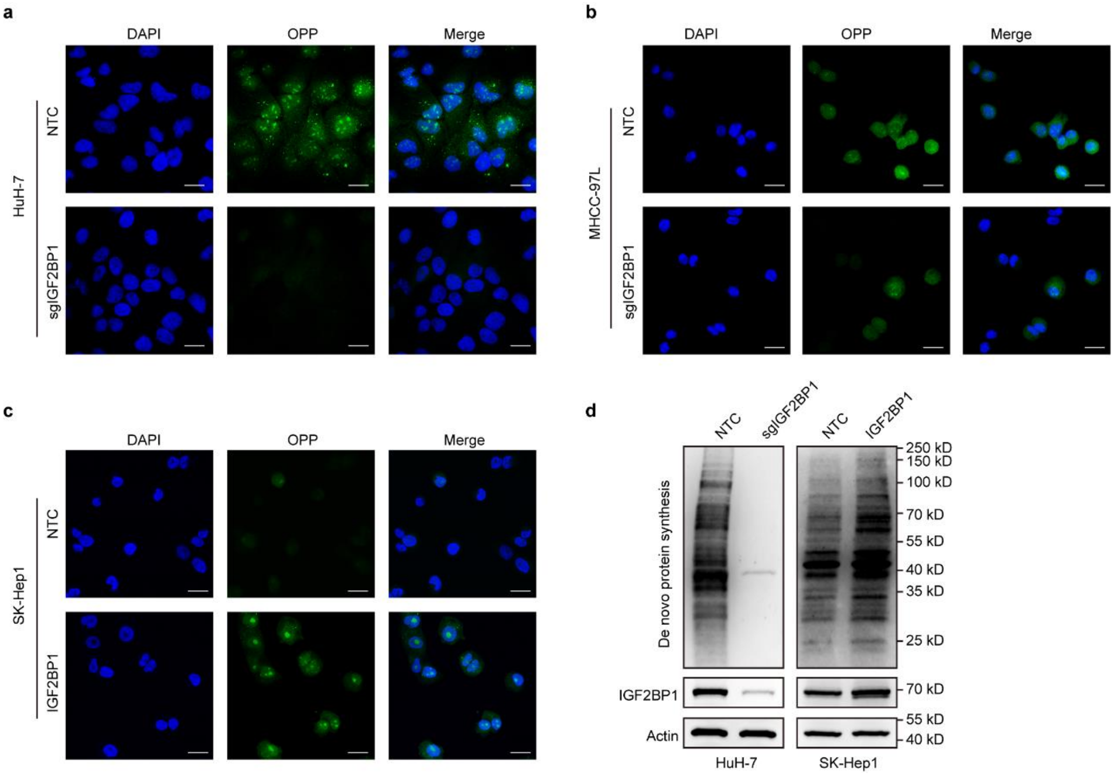

Figure S6. IGF2BP1 enhances mRNA translation in HCC cells.

(a-c) Representative immunofluorescent images of OP-Puro assays to quantify the amounts of nascent peptides in HuH-7 **(a)** and MHCC-97L **(b)** cells with IGF2BP1 knockout or SK-Hep1 cells with IGF2BP1 overexpression **(c)**. Scale bar, 50 µm. **(d)** Representative Western blotting images of SUnSET assays to quantify the amounts of nascent peptides in HuH-7 (left) cells with IGF2BP1 knockout or SK-Hep1 (right) cells with IGF2BP1 overexpression.

Supplementary Figure 7.

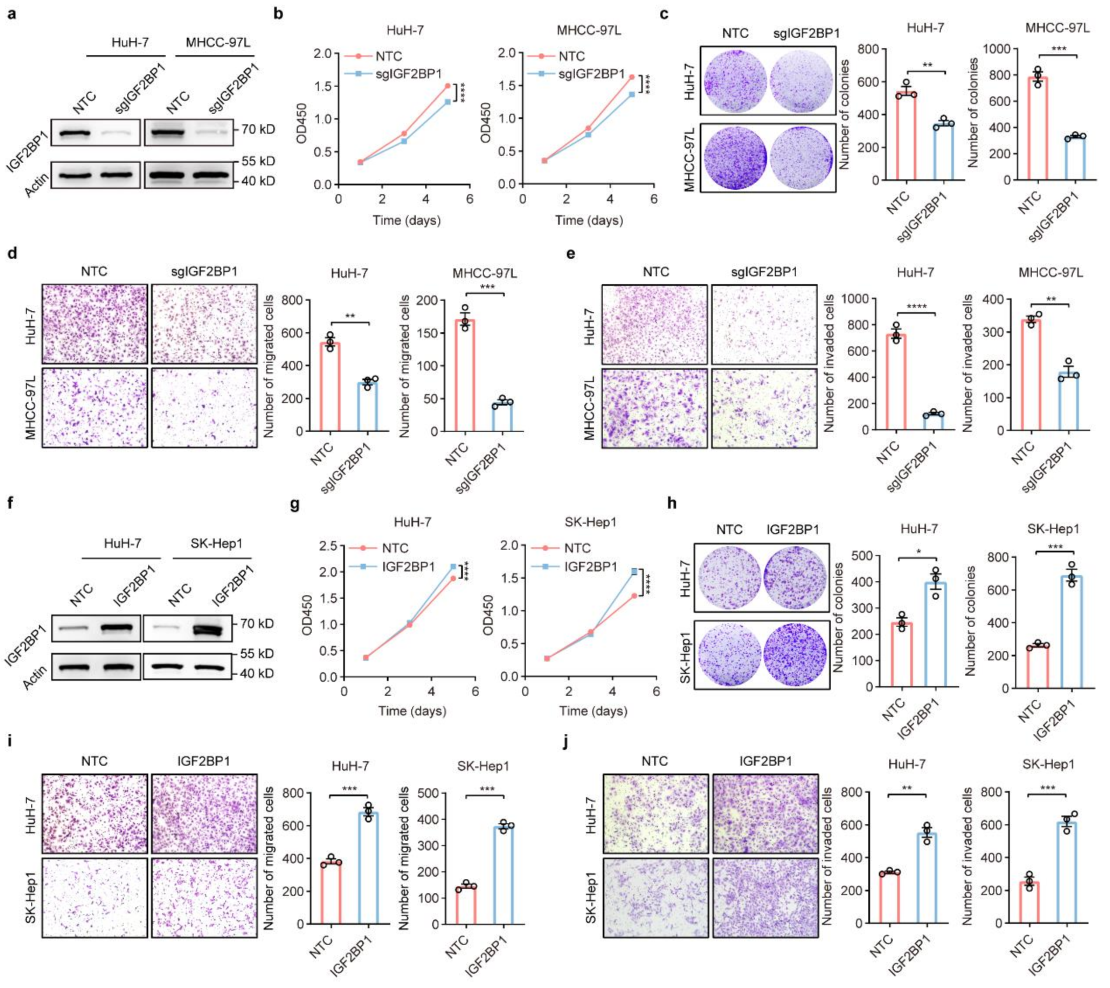

Figure S7. IGF2BP1 accelerates HCC cell proliferation, migration and invasion.

(a) Western blot analysis of IGF2BP1 expression in control or IGF2BP1-knockout HuH-7 and MHCC-97L cells. **(b)** The effect of IGF2BP1 knockout on cell viability in HuH-7 and MHCC-97L cells. Data are represented as mean ± SEM (n = 3). Two-way ANOVA with Tukey's multiple comparisons test. **(c)** Colony formation assay: representative colony images of HuH-7 and MHCC-97L cells with or without IGF2BP1 knockout (left) and statistical histogram of colonies from three replicates (right). Data are represented as mean ± SEM (n = 3). Unpaired Student's t-test. **(d)** Transwell migration assay: representative migration images of HuH-7 and

MHCC-97L cells with or without IGF2BP1 knockout (left) and statistical histogram of migration cells from three replicates (right). Data are represented as mean ± SEM (n = 3). Unpaired Student’s t-test. **(e)** Transwell invasion assay: representative invasion images of HuH-7 and MHCC-97L cells with or without IGF2BP1 knockout (left) and statistical histogram of invasion cells from three replicates (right). Data are represented as mean ± SEM (n = 3). Unpaired Student’s t-test. **(f)** Western blot analysis of IGF2BP1 expression in control or IGF2BP1-overexpressing HuH-7 and SK-Hep1 cells. **(g)** The effect of IGF2BP1 overexpression on cell viability in HuH-7 and SK-Hep1 cells. Data are represented as mean ± SEM (n = 3). Two-way ANOVA with Tukey's multiple comparisons test. **(h)** Colony formation assay: representative colony images of HuH-7 and SK-Hep1 cells with or without IGF2BP1 overexpression (left) and statistical histogram of colonies from three replicates (right). Data are represented as mean ± SEM (n = 3). Unpaired Student’s t-test. **(i)** Transwell migration assay: representative migration images of HuH-7 and SK-Hep1 cells with or without IGF2BP1 overexpression (left) and statistical histogram of migration cells from three replicates (right). Data are represented as mean ± SEM (n = 3). Unpaired Student’s t-test. **(j)** Transwell invasion assay: representative invasion images of HuH-7 and SK-Hep1 cells with or without IGF2BP1 overexpression (left) and statistical histogram of invasion cells from three replicates (right). Data are represented as mean ± SEM (n = 3). Unpaired Student’s t-test. *$p < 0.05$, **$p < 0.01$, ***$p < 0.001$, ****$p < 0.0001$.

Supplementary Figure 8.

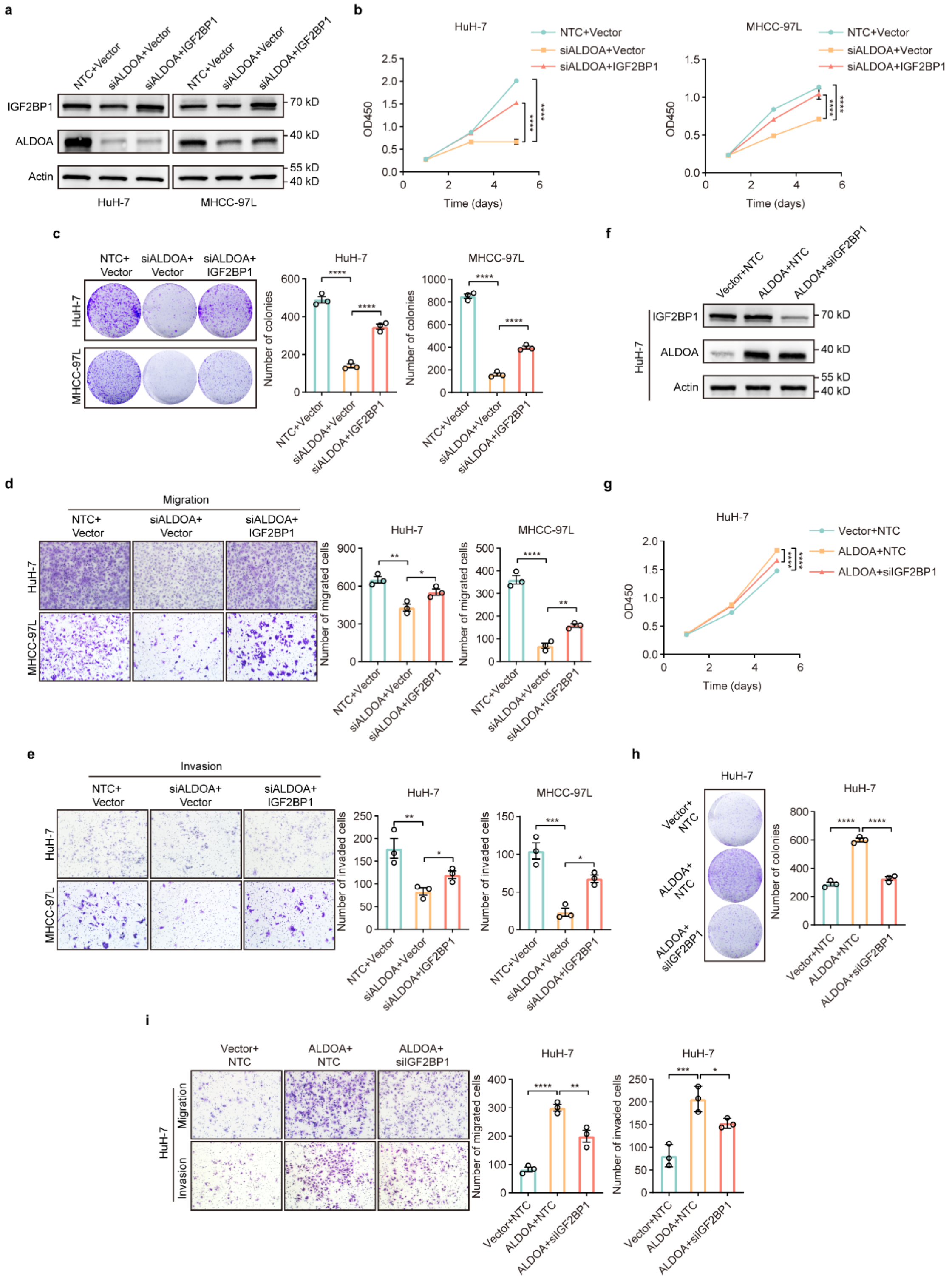

Figure S8. Ectopic expression of IGF2BP1 strongly abolished the tumor suppressive effect of ALDOA deficiency in HCC.

(a) Rescued expression of IGF2BP1 in HuH-7 and MHCC-97L cells upon ALDOA knockdown. **(b)** Rescued effects of forced expression of IGF2BP1 on cell viability of HuH-7 and MHCC-97L cells upon ALDOA knockdown. Data are represented as mean ± SEM (*n* = 3). Two-way ANOVA with Tukey's multiple comparisons test. **(c)** Rescued effects of forced expression of IGF2BP1 on colony formation abilities of HuH-7 and MHCC-97L cells upon ALDOA knockdown. Data are represented as mean ± SEM (*n* = 3). One-way ANOVA with Dunnett's multiple comparisons test. **(d)** Rescued effects of forced expression of IGF2BP1 on migration of HuH-7 and MHCC-97L cells upon ALDOA knockdown. Data are represented as mean ± SEM (*n* = 3). One-way ANOVA with Dunnett's multiple comparisons test. **(e)** Rescued effects of forced expression of IGF2BP1 on invasion of HuH-7 and MHCC-97L cells upon ALDOA knockdown. Data are represented as mean ± SEM (*n* = 3). One-way ANOVA with Dunnett's multiple comparisons test. **(f)** ALDOA and IGF2BP1 protein expression after knockdown of IGF2BP1 in ALDOA-overexpressing HuH-7 cells. **(g)** Cell viability of ALDOA-overexpressing HuH-7 cells with or without IGF2BP1 depletion. Data are represented as mean ± SEM (*n* = 3). Two-way ANOVA with Tukey's multiple comparisons test. **(h)** Colony formation assay: representative colony images of ALDOA-overexpressing HuH-7 cells with or without IGF2BP1 depletion (left) and statistical histogram of colonies from three replicates (right). Data are represented as mean ± SEM (*n* = 3). One-way ANOVA with Dunnett's multiple comparisons test. **(i)** Transwell migration and invasion assay: representative migration and invasion images of ALDOA-overexpressing HuH-7 cells with or without

IGF2BP1 depletion (left) and statistical histogram of migration and invasion cells from three replicates (right). Data are represented as mean ± SEM (n = 3). One-way ANOVA with Dunnett's multiple comparisons test. *$p < 0.05$, **$p < 0.01$, ***$p < 0.001$, ****$p < 0.0001$.

Supplementary Figure 9.

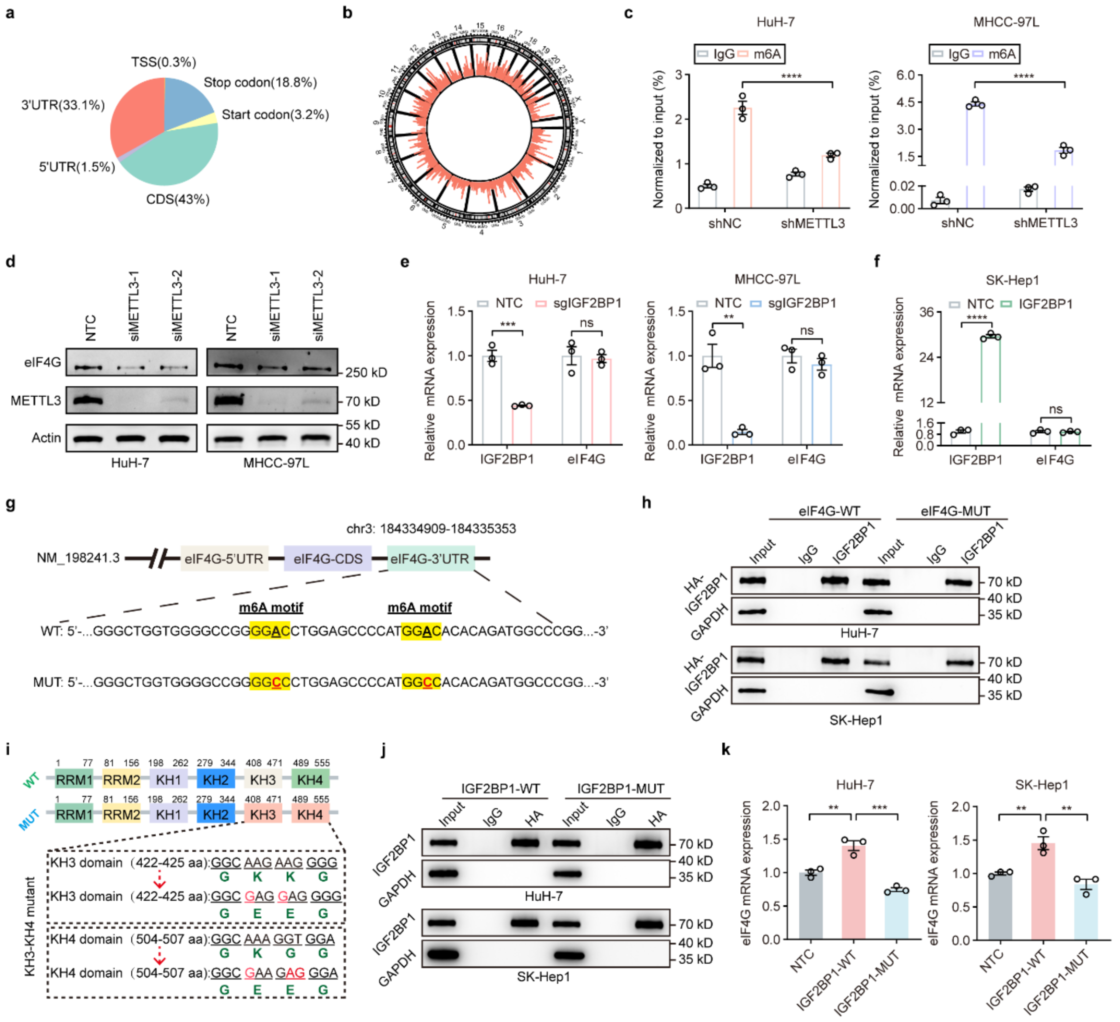

Figure S9. IGF2BP1 recognizes the m^{6}A modification in eIF4G-3'UTR and regulates its expression.

(a) Distribution of m^{6}A sites within different gene regions. **(b)** Circos plot demonstrating the distribution of m^{6}A peaks in the human transcriptome of HuH-7 cells. **(c)** MeRIP-qPCR was performed in HuH-7 and MHCC-97L cells with or without METTL3 knockdown. Data are represented as mean ± SEM (n = 3). Two-way ANOVA with Tukey's multiple comparisons test. **(d)** Western blot analysis of eIF4G expression in METTL3 knockdown or control HuH-7 and MHCC-97L cells. **(e-f)**

RT-qPCR analysis of eIF4G expression in IGF2BP1 knockout HuH-7 and MHCC-97L cells **(e)** or IGF2BP1 overexpression SK-Hep1 cells **(f)**. Data are represented as mean ± SEM (*n* = 3). Unpaired Student's t-test. **(g)** A schematic presentation of the pmir-GLO luciferase reporters containing WT and MUT (GGAC to GGCC) eIF4G mRNA 3′UTR. **(h)** Western blot confirmed the IGF2BP1 IP in eIF4G-WT or eIF4G-MUT overexpressing HuH-7 and SK-Hep1 cells. **(i)** Schematic structures demonstrating RNA binding domains within IGF2BP1 protein and a summary of IGF2BP1 variants utilized in this study. **(j)** Western blot confirmed the IGF2BP1-HA IP in IGF2BP1-WT or IGF2BP1-MUT overexpressing HuH-7 and SK-Hep1 cells. **(k)** RT-qPCR analysis of eIF4G expression in IGF2BP1-WT or IGF2BP1-MUT overexpressing HuH-7 and SK-Hep1 cells. Data are represented as mean ± SEM (*n* = 3). One-way ANOVA with Dunnett's multiple comparisons test. ns, not significant, **$p < 0.01$, ***$p < 0.001$, ****$p < 0.0001$.

Supplementary Figure 10.

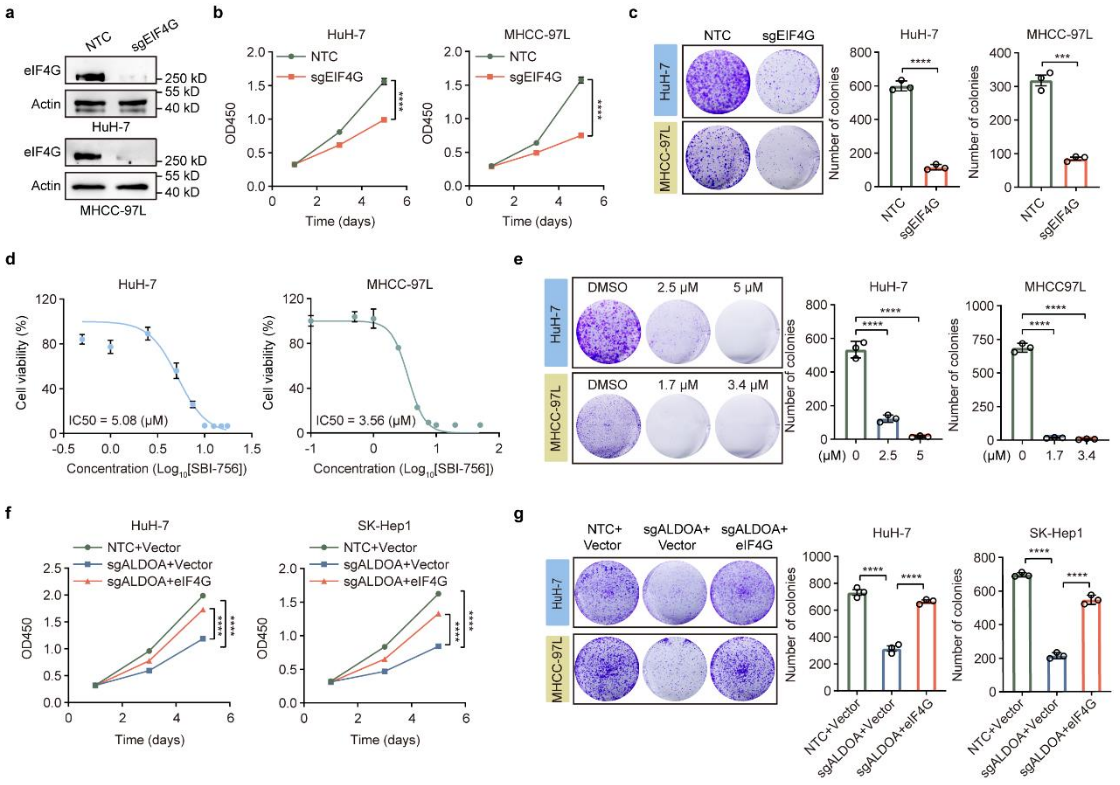

Figure S10. eIF4G mediates the effects of ALDOA in HCC cells.

(**a**) Western blot analysis of eIF4G expression in control or eIF4G-knockout HuH-7 and MHCC-97L cells. **(b)** The effect of eIF4G knockout on cell viability in HuH-7 and MHCC-97L cells. Data are represented as mean ± SEM (*n* = 3). Two-way ANOVA with Tukey's multiple comparisons test. **(c)** Colony formation assay: representative colony images of HuH-7 and MHCC-97L cells with or without eIF4G knockout (left) and statistical histogram of colonies from three replicates (right). Data are represented as mean ± SEM (*n* = 3). Unpaired Student’s t-test. **(d)** The effect of eIF4G inhibitor-SBI756 on cell viability in HuH-7 and MHCC-97L cells. **(e)** Colony formation assay: representative colony images of HuH-7 and MHCC-97L cells with or without eIF4G inhibitor-SBI756 treatment (left) and statistical histogram of

colonies from three replicates (right). Data are represented as mean ± SEM (n = 3). One-way ANOVA with Dunnett's multiple comparisons test. **(f)** CCK-8 assays showing the effect of eIF4G on restoring cell viability in ALDOA-knockout HuH-7 and SK-Hep1 cells. Data are represented as mean ± SEM (n = 3). Two-way ANOVA with Tukey's multiple comparisons test. **(g)** Colony formation assay: representative colony images of ALDOA-knockout HuH-7 and MHCC-97L cells with or without eIF4G overexpression (left) and statistical histogram of colonies from three replicates (right). Data are represented as mean ± SEM (n = 3). One-way ANOVA with Dunnett's multiple comparisons test. ***$p < 0.001$, ****$p < 0.0001$.

Supplementary Figure 11.

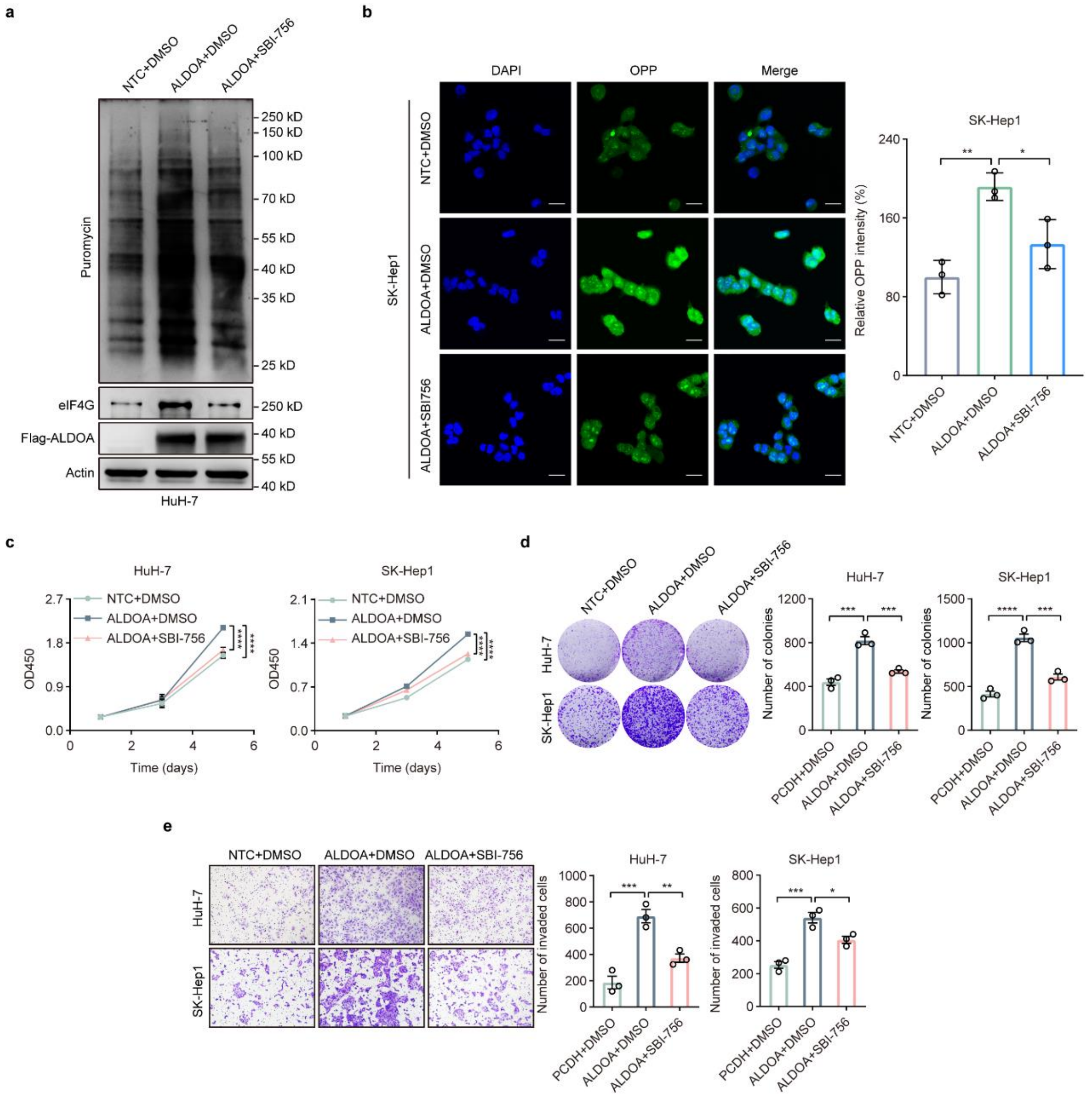

Figure S11. eIF4G inhibitor SBI-756 impaired the promotion effect of ALDOA overexpression on protein biosynthesis, HCC cell growth and invasion in vitro.

(**a**) Western blot analysis of puromycin incorporated into nascent peptides to monitor global mRNA translation in ALDOA-overexpressing HuH-7 cells after treatment with SBI-756 or vehicle, respectively. Actin was detected as a loading control. (**b**) OP-Puro analysis showing that the increase in protein synthesis induced by ALDOA overexpression was attenuated by SBI-756 treatment in SK-Hep1 cells. Scale bar, 50

μm. Data are presented as mean ± SD ($n = 3$). One-way ANOVA with Dunnett's multiple comparisons test. (**c-e**) CCK-8 proliferation assay (**c**), colony formation assay (**d**), and Transwell invasion assay (**e**) were performed in ALDOA-overexpressing HuH-7 and SK-Hep1 cells treated with the eIF4G inhibitor SBI-756 (5 μm) or vehicle. Data are presented as mean ± SD ($n = 3$). Two-way ANOVA with Tukey's multiple comparisons test were performed for (**c**); one-way ANOVA with Dunnett's multiple comparisons test were performed for (**d, e**). *$p < 0.05$, **$p < 0.01$, ***$p < 0.001$, ****$p < 0.0001$.

Table S6. The sequences of siRNAs and sgRNAs used in this study.

Name	Sequences (5'-3')	Note
siALDOA-1	GGCGTTGTGTGCTGAAGAT	siRNA
siALDOA-2	CCATCAACCTCAATGCCAT	sequences
siIGF2BP1-1	GGCTCAGTATGGTACAGTA	
siIGF2BP1-2	TGAAGATCCTGGCCCATAA	
sgALDOA-F	CACCGCCAACAGGTAGACAAGGGCG	sgRNA
sgALDOA-R	AAACCGCCCTTGTCTACCTGTTGGC	sequences
sgIGF2BP1-F	CACCGTGAATGTCACCTATTCCAAC	
sgIGF2BP1-R	AAACGTTGGAATAGGTGACATTCAC	
sgEIF4G-F	CACCGTGGTGCATTCGTCGCTGAAC	
sgEIF4G-R	AAACGTTCAGCGACGAATGCACCAC	

Table S7. Antibodies used in this study.

Antibodies	Source	Identifier
GAPDH	Proteintech	Cat# 60004-1-Ig
Actin	Proteintech	Cat# 66009-1-Ig
ALDOA	Proteintech	Cat# 67453-1-Ig
ALDOA	Proteintech	Cat# 11217-AP
ALDOA	Bethyl	Cat# A304-495A
IGF2BP1	Proteintech	Cat# 22803-1-AP
eIF4G	CST	Cat# 2469S
Ki67	Proteintech	Cat# 27309-1-AP
FLAG	Proteintech	Cat# 20543-1-AP
HA	Proteintech	Cat# 66006-2-Ig
Goat anti-Mouse IgG (H+L), HRP conjugate	Proteintech	Cat# SA00001-1
Goat anti-Rabbit IgG (H+L), HRP conjugate	Proteintech	Cat# SA00001-2
Rabbit Control IgG	CST	Cat# 2729
Puromycin	Millipore	Cat# MABE343
